# Microfluidics to Meet Antibiotic Resistance: A Growing Research Frontier

**DOI:** 10.3390/antibiotics14121232

**Published:** 2025-12-07

**Authors:** Mikhail Y. Zhitlov, Vladimir A. Korshun, Vera A. Alferova

**Affiliations:** Shemyakin-Ovchinnikov Institute of Bioorganic Chemistry, Miklukho-Maklaya 16/10, 117997 Moscow, Russia; droplbox38@gmail.com (M.Y.Z.); v-korshun@yandex.ru (V.A.K.)

**Keywords:** antibiotic resistance, microfluidics, drug screening, single-cell imaging

## Abstract

Antimicrobial resistance remains one of the most urgent challenges in modern medicine, demanding innovative research tools for understanding and combating bacterial adaptation. Microfluidic technologies enable precise control over experimental conditions, single-cell resolution, and high-throughput analysis, offering unique advantages over traditional microbiological methods. This review summarizes recent (2020–2025) developments in the application of microfluidics to antibiotic resistance research, emphasizing approaches used in fundamental studies rather than diagnostic implementations. The discussed technologies are grouped according to their primary research focus: (i) microfluidic cultivation and screening of antibiotic-producing microorganisms; (ii) tools for antibiotic screening and mechanistic studies, and (iii) models for studying microbial stress responses and resistance development. Collectively, these approaches provide unprecedented insight into antibiotic action, resistance evolution, and microbial physiology. Continued development and integration of microfluidics with complementary analytical tools will further accelerate the discovery of novel antimicrobials and rational design of combination therapies, ultimately bridging the gap between fundamental microbiology and translational applications in antimicrobial resistance research.

## 1. Introduction

Microfluidic technologies have an extremely wide range of applications and have revolutionized many fields: organic [1,2] and nanoparticle [3,4] synthesis; drug discovery [5,6], development [7], and delivery [8,9]; production of RNA- and DNA-loaded lipid nanoparticles for gene delivery [10,11,12]; reproductive technologies [13,14]; live-cell delivery [15]; CAR-T-cell immunotherapy [16,17]; cell separation [18] and other optical sorting [19]; environmental analysis [20]; aging and rejuvenation studies [21]; biomarker diagnostics [22]; food quality analysis [23,24] and biosensing [25,26]; organ-on-chip [27,28] and 3D-cell-culture [29,30] models; single-cell analysis [31,32] (including cancer research [33]) and cell nanoencapsulation [34]; protein screening [35]; protein interaction studies [36]; and many others.

Antibiotic resistance is a complex environmental phenomenon [37,38] recognized as an increasing global threat to public health [39,40,41]. Naturally, this huge problem increasingly attracts the power of various areas of microfluidic technologies, especially breakthrough droplet ones. Related areas of microfluidics have been extensively reviewed previously; specific review articles in the past five years were devoted to droplet microfluidics [31,42] (including general aspects [43,44], equipment [45,46], pharmaceutical applications [47], and bacterial cultivation [42,48]) and microbiological single-cell manipulations [49,50] and analysis [51,52]. Specific applications of microfluidic technologies, including antimicrobial susceptibility testing and resistance development studies [53,54], bacterial patterning [55], synthetic microbial communities construction [56,57,58] and biomass processing [59,60], biofilm investigation [61], and environmental studies [62,63,64], have also been recently reviewed.

The objective of this review is to provide a concise yet comprehensive overview of recent (2020–2025) applications of microfluidic technologies that contribute to addressing the pressing issue of antimicrobial resistance. By summarizing key experimental designs across various application areas, this work aims to serve as a valuable foundation for future research in the field. The review focuses primarily on microfluidic approaches used in fundamental studies of antibiotic resistance. Newly developed technologies, as well as successful examples of adaptation or refinement of existing methods, are organized into three major sections according to the principal biological question addressed. The first section highlights studies on microbial strains that produce antimicrobial compounds, emphasizing bioactive molecules and antibiotics themselves. The second section summarizes microfluidic approaches for antibiotic screening and mechanistic investigations. Finally, as overcoming resistance and identifying novel targets depends on a detailed understanding of microbial physiology, the third section reviews methods for studying the emergence of resistance and the stress responses of model microorganisms.

The widespread use of antibiotics in the food and agricultural industries significantly contributes to the spread of antibiotic resistance, leading to the accumulation of antibiotics in the environment and creating evolutionary pressure for the selection of resistant isolates. Microfluidic technologies are used to detect antibiotics in food products and other complex matrices [65,66,67], but this area is only indirectly related to antibiotic resistance and is therefore outside the scope of this research.

Specific microfluidic models simulating infection conditions are also being developed to study infectious agents in clinical practice. For example, Microfluidic Silicon Membrane Canalicular Arrays (μSiM-CA) [68] were developed to investigate the colonization of the osteocyte lacuno-canalicular network of cortical bone by *Staphylococcus aureus*. Despite the importance of these approaches to medicine, they are not directly related to the problem of antibiotic resistance and are therefore also not discussed in this review. One of the most developed fields of microfluidic devices application for clinical analysis is antibiotic susceptibility testing (AST), enabling rapid identification of suitable personalized therapeutic strategies for infectious diseases. These methods were extensively reviewed previously [69,70,71,72,73,74,75,76,77,78,79,80,81,82] and are not included in the scope of this review. Nonetheless, AST techniques require the development of specific and sensitive growth detection approaches, along with effective live/dead sorting, so the most prominent examples with high potential in the field of antibiotic resistance studies are selectively discussed.

## 2. General Overview of Microfluidic Devices and Approaches

This review aims to outline practical microfluidic solutions suitable for application in antimicrobial resistance research, exemplified by the most recent works in the field. To establish a common framework for the subsequent sections, we begin with a brief introduction to essential microfluidic concepts, widely used device architectures, and commonly applied fabrication techniques and materials.

### 2.1. Approaches to Microfluidic Device Fabrication

Microfluidics refers to the manipulation of fluids at the micrometer scale, typically from a few to several hundred micrometers [83]. Such dimensions enable precise spatial and temporal control over the chemical and physical environment, which is why microfluidic technologies are applied in engineering, biology, chemistry, physics, etc. Despite their advantages, microfluidic systems face intrinsic challenges, most notably the need for highly precise geometries and careful selection of materials that support mechanical stability, chemical compatibility, and biological functionality.

Microfluidic devices typically possess features at the tens-of-microns scale. Consequently, their fabrication strategies parallel microelectromechanical systems (MEMS) technologies, which have recently become widespread in consumer electronics, such as inertial sensors, pressure sensors, and microphones. Replica molding is among the most widely adopted approaches, in which a master mold, produced via micro-cutting, laser ablation, electron beam machining, etc., is replicated using polydimethylsiloxane (PDMS) soft lithography or via thermoplastic molding [84]. Alternatively, such devices can be fabricated using direct fabrication techniques, including glass etching, polymer micromachining, and additive manufacturing. Broadly speaking, microfabrication strategies for microfluidic devices fall into two categories: additive and subtractive approaches. Additive methods, such as photolithography, 3D printing, and two-photon polymerization, allow for the construction of three-dimensional structures via layer-wise material deposition, whereas subtractive methods, such as laser ablation or micromachining, remove material to form microchannels and features.

The most common additive method is photolithography, whereby the master mold serves solely as a template for constructing the final microfluidic device. SU-8 photoresist on silicon substrates enables the fabrication of microstructures with heights of up to 1 mm and aspect ratios (between height and width of features) up to 40:1 [85]. Alternative photopolymers, such as PMMA (polymethylmethacrylate), combined with X-ray lithography, allow for even higher aspect ratios. Photolithography provides high resolution (~1 micron), dimensional stability, and excellent reproducibility [86]. Moreover, multilayer structures can be achieved by sequential deposition and patterning of photoresist layers. The major limitation of this method is the need for specialized facilities, including cleanrooms and high-precision optical instrumentation.

Another additive manufacturing method, 3D printing, has become an accessible alternative for prototyping both molds and devices. Technologies such as selective laser sintering and fused deposition modeling support a broad range of materials, including biodegradable polylactic acid, chemically inert polypropylene and polyethylene, and high-temperature polymers, such as polycarbonate and polyarylether ketones (PAEKs). Typical feature sizes are around 100 microns, limited by the nozzle and mechanical precision [84]. SLA 3D printing, based on layer-by-layer photopolymerization using UV lasers, LCD mask, or digital light projection (DLP) chips, offers cost-effectiveness and higher precision (down to 10 microns), but lacks the choice of biocompatible polymers. The accessibility of modern desktop 3D printers can further expand their use in microfluidics research.

Subtractive manufacturing techniques, such as milling, turning, grinding, electric-discharge machining (EDM), and laser ablation, offer additional fabrication routes, although not all of them are suitable for the microscale features required in microfluidics. Micromachining using high-speed CNC milling can generate structures with feature sizes down to approximately 25 µm using ultra-small cutting tools and high-precision positioning systems. EDM enables the fabrication of complex, high-aspect-ratio, and even non-planar geometries [87] across a diverse range of materials, including metals [84]. However, this approach is limited by tool fragility, rapid wear, and the use of extremely small end mills. Laser-based fabrication, including femtosecond laser inscription, supports rapid prototyping in glass and polymers with sub-micron accuracy, though it requires costly instrumentation [88].

### 2.2. Materials for Device Fabrication

The methods described above can be applied to direct chip fabrication; however, they strictly limit suitable materials. Therefore, they are mostly used to manufacture master molds. Common device fabrication materials are summarized in dedicated reviews [89,90]. The most widely applied material for replica molding is polydimethylsiloxane (PDMS) [91], due to the excellent optical clarity of the material, its biocompatibility, and ease of bonding. PDMS also enables the fabrication of multilayer devices incorporating microvalves and thin membranes. However, PDMS has inherent drawbacks: it is permeable to gases and tends to absorb small hydrophobic molecules, such as drugs, hormones, dyes, and solvents [92]. Other disadvantages include unstable surface treatment, bonding limitations, and leaching of cytotoxic oligomers [93]. These properties can introduce experimental artifacts, especially in antimicrobial assays.

Thermoplastic materials—including cyclic olefin copolymer (COC), cyclic olefin polymer (COP), polycarbonate (PC), and polystyrene (PS)—provide an attractive alternative. Fabrication techniques such as injection molding and hot embossing enable high-throughput production of durable microfluidic devices with excellent reproducibility [91]. Thermoplastics exhibit improved chemical resistance and reduced adsorption of hydrophobic compounds compared to PDMS, though they are generally less gas-permeable and more expensive for prototyping.

Other frequently applied material is glass, which offers outstanding chemical resistance, optical clarity, and low analyte adsorption, making it highly suitable for applications requiring thermal or chemical robustness. Glass devices are commonly fabricated through wet etching, laser processing, or the bonding of patterned wafers. However, fabrication is more labor-intensive and often requires high-temperature bonding steps.

### 2.3. Microfluidics Experimental Setups

Microfluidic systems are commonly divided into two major conceptual categories: droplet microfluidics and continuous-flow (or “single-phase”) microfluidics. This distinction is widely used and remains meaningful, although each category encompasses numerous subtypes as well as hybrid architectures. In practice, the choice of microfluidic format is dictated primarily by the biological question being addressed.

Continuous-flow microfluidics is the manipulation of liquid flow through fabricated microchannels without breaking continuity. In these devices, laminar flow dominates, enabling stable gradients, predictable mass transport, and the controlled exposure of cells or reagents to defined conditions. Channel geometry, flow rate, and material properties collectively shape the microenvironment, making continuous-flow platforms well-suited for long-term cultivation, real-time imaging, and studies requiring tightly regulated hydrodynamics. This class of microfluidic systems spans a broad range of architectures, from simple straight channels to complex networks incorporating traps, mixers, valves, or perfusion chambers.

**Figure 1:**
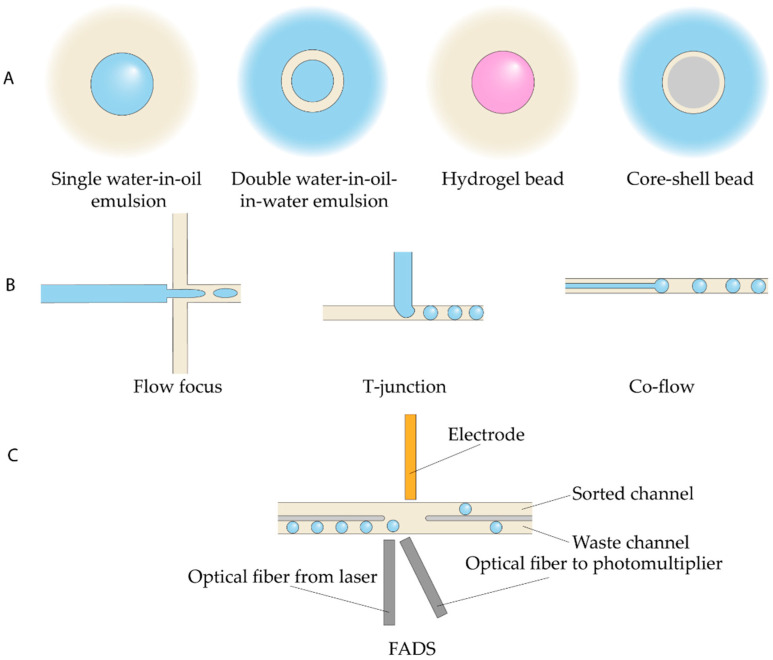
Key features of droplet microfluidics. (**A**) Common emulsion types, (**B**) typical droplet-generating microfluidic geometries, (**C**) schematic of a fluorescence-activated droplet sorting (FADS) chip.

Droplet microfluidic (discrete-phase microfluidics) involves the generation, manipulation, and analysis of discrete, compartmentalized microreactors (droplets, vesicles, hydrogel beads) with precisely controlled volume and composition, dispersed in an immiscible carrier fluid (typically mineral or fluorinated oil) [94] (Figure 1). Various emulsion formats can be produced, including single emulsions, multiple emulsions, core–shell droplets, and hydrogel beads (Figure 1A). Droplets are typically generated using one of three common passive geometries—co-flow, cross-flow, or flow-focusing (Figure 1B)—each of which allows reproducible droplet formation over a wide range of volumes and generation frequencies (Table 1) [45]. Despite high-generation throughput, emulsification protocols are still limited in yield: typical devices produce up to 1 mL of emulsion per hour, limiting scaled applications [95].

Droplet sorting and analysis based on their absorbance, optical density, luminosity, or conductivity requires specialized methods, including Fluorescence-Activated Cell Sorting (FACS), Fluorescence-Activated Droplet Sorting (FADS), Pneumatic-Based Droplet Sorting, Dielectrophoretic-Based Droplet Sorting, Acoustic-Based Droplet Sorting, etc. [96]. Conventional cell sorters are not compatible with water-in-oil emulsions, necessitating the implementation of water-in-oil-in-water double emulsions or beads, which are often impractical. Specifically, FADS was developed to overcome this limitation (Figure 1C). The FADS method may rely on dielectrophoresis, acoustic actuation, or pneumatic microvalves to deflect droplets in real time, enabling high-throughput sorting directly within the microfluidic environment. Unlike traditional FACS instruments, FADS allows direct visualization of individual sorting events with high-speed cameras, providing a powerful tool for droplet-based screening workflows, but requires sophisticated custom-built equipment.

**Table 1:** Throughput of droplet generation and sorting devices.

Method	Frequency	Droplet Size	Ref
Droplet generation methods			
Flow-focusing generator	10–10,000 Hz	10–100 μm	[97]
T-junction	10–100 Hz	50–500 μm	
Step emulsification	100–10,000 Hz	<100 μm	
Co-flow	Up to 100 kHz	Up to 500 μm	[98]
Droplet sorting methods			
FACS	up to 1 MHz	up to 200 μm	[99]
FADS	30 kHz	depends on channel sizes	[100]

Both types of microfluidic systems are highly susceptible to clogging, one of the most common and disruptive failure modes in microscale devices. Because microfluidic channels operate with small cross-sectional dimensions and laminar flow, even sub-micron particles or aggregates can obstruct channels, destabilize flow profiles, or halt device operation entirely. In droplet generation, even partial occlusion can significantly affect flow rates and droplet uniformity. Clogging typically results from several mechanisms: particulate contamination such as dust, undissolved solutes, or cell aggregates; biofouling, including adsorption of proteins or bacterial attachment and proliferation; droplet adhesion or coalescence in droplet microfluidics due to wetting instability or surfactant depletion; precipitation of salts or poorly soluble compounds; and bubble formation from dissolved gases or temperature fluctuations. The most appropriate mitigation strategies depend strongly on device architecture and experimental conditions and are summarized in dedicated reviews [101].

### 2.4. Flow Control Strategies

Precise regulation of fluid flow is essential for achieving reproducible microfluidic operation, particularly in droplet-based assays used in antimicrobial resistance research. Several flow-control strategies are employed, each offering distinct advantages in terms of stability, responsiveness, cost, and compatibility with biological assays.

Syringe pump-based flow control remains one of the most commonly used approaches due to its simplicity, commercial availability, and compatibility with a wide range of materials. Syringe pumps provide highly stable, low-pulsation volumetric flow rates and are widely used for droplet generation, perfusion assays, and long-term culturing. However, they exhibit slow response times and are prone to drift in multi-hour experiments, especially when multiple pumps must be synchronized. Their footprint also limits portability and point-of-care integration [102].

Pressure-driven flow control systems—typically based on compressed air or nitrogen applied to fluid reservoirs—enable rapid and precise control of flow rates and shear stresses. Modern pressure controllers equipped with integrated feedback loops achieve sub-millibar resolution and millisecond-scale response times, facilitating highly stable droplet generation and rapid switching between reagents [103]. Pressure-driven flow also reduces pulsation and dead volume compared with syringe pumps. Despite their advantages, these systems require rigid reservoirs and stable gas supplies, which may complicate use outside laboratory settings.

Vacuum-driven flow represents an alternative strategy, particularly suitable for portable applications and devices using capillary valves [104]. By applying negative pressure at the outlet, vacuum-driven systems eliminate the need for pressurized reagents and can operate using simple syringe- or pump-based vacuum sources. However, vacuum-based setups are more sensitive to leak formation and typically offer less precise flow regulation than positive-pressure systems.

Centrifugal microfluidics (“lab-on-a-disc”) employs rotational force to drive fluid motion, enabling valving, metering, mixing, and droplet production without external pumps [105]. Flow rates depend on rotational speed, disc geometry, and fluid properties. This strategy facilitates highly portable and low-cost devices, including diagnostic platforms, but provides limited real-time modulation of flow once the disc is sealed and spinning.

## 3. Producing Strains: Discovery and Cultivation

Many ecological processes, including the production of secondary metabolites, arise from single-cell behaviors and interaction-dependent regulation. At present, natural microbial consortia are mainly explored through cultivation-based approaches, which limit the number of strains that can be screened and often provide a biased view of microbial diversity [32]. Microfluidic technologies—particularly droplet-based systems—have emerged as powerful tools for single-cell studies [32,42], including the discovery and development of biocontrol agents and antibiotic-producing microorganisms.

**Figure 2:**
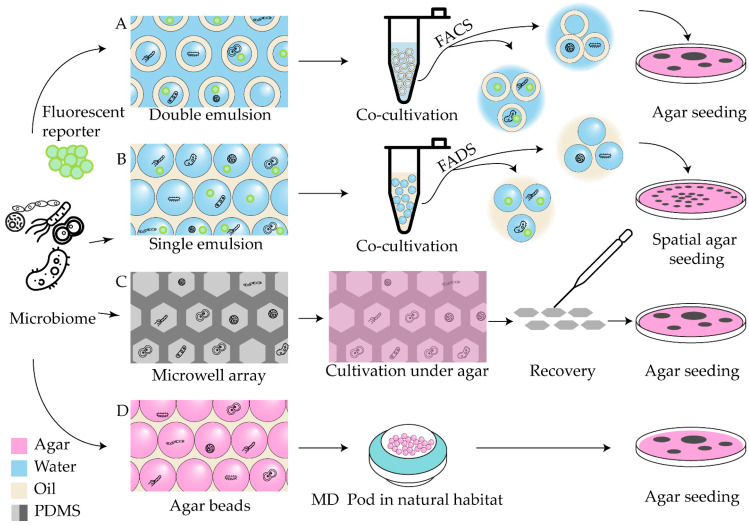
Schematic representation of microfluidic approaches for natural biodiversity profiling and antibiotic producer discovery. (**A**) Cultivation of bacteria in double emulsions. Droplets are generated by flow-focusing. Chips are PDMS replicas of SU-8/silicon master molds fabricated by photolithography and bonded to glass slides. PDMS surfaces are modified with PVA, Mowiol, trichloro(octadecyl)silane, or Aquapel. Sorting is performed by FACS. (**B**) Cultivation of bacteria in single emulsions with subsequent spatial seeding. Droplets are generated using a T-junction. Chips are PDMS replicas of SU-8/silicon master molds bonded to glass slides. PDMS is modified with Novec 1720. Sorting is performed by FADS. (**C**) Microwell-array–based cultivation. The microwell array is formed from PDMS replicas of SU-8/silicon soft-lithography master molds. (**D**) MD Pod platform. Agar beads are produced by cross-flow droplet generation in an SLA 3D-printed chip. The MD Pod cultivation chamber is fabricated by two-photon polymerization.

One of the most notable examples of a microfluidic platform for biodiversity screening involves single-cell cultivation of microorganisms in double water-in-oil-in-water (W/O/W) emulsions, followed by fluorescence-activated cell sorting (FACS), as described previously [106] (Figure 2A). This approach was recently enhanced through the integration of live biosensors, allowing for precise monitoring of antimicrobial activity [107]. This platform has been applied to functional profiling of diverse microbial communities, enabling the identification of amicoumacin-producing strains [108], screening of wild-animal microbiota for potential biocontrol agents [109], and isolation of a novel bacteriocin active against methicillin-resistant *S. aureus* (MRSA) [110]. A similar concept underlies the GrowMIDE platform, developed for studying human gut microbiota, which demonstrated the ability to recover rare microbial species [111]. The key advantage of such double-emulsion systems lies in their compatibility with conventional flow cytometry equipment, enabling effective enrichment of bacterial populations containing bioactive isolates through fluorescence-gated sorting. Gel microbeads share this advantage with W/O/W emulsions; a recent proof-of-concept study demonstrated their application for high-throughput screening of antibiotic-producing strains using FACS-based sorting [112].

Selective isolation of individual colonies of interest for further cultivation and analysis remains particularly challenging in most microfluidic systems. A droplet-based microscale cultivation platform was developed to enable high-throughput screening of antimicrobial activity prior to strain isolation [113]. In this approach, the microbial community is encapsulated into water-in-oil (W/O) droplets, which are incubated in bulk and subsequently injected into a 100 μm capillary for deposition onto an agar surface (Figure 2B). This results in spatially separated microcolonies derived from individual droplets. Although the current method requires specialized equipment, related techniques for automated microdroplet dispensing with single-colony resolution are under active development [114].

An alternative on-chip cultivation approach, termed the digital plating (DP) platform, was proposed to simplify single-cell isolation [115]. The DP workflow involves discretizing the sample into a high-density picoliter microwell array (PicoArray device), followed by coverage with a thin agar medium sheet for incubation and subsequent microscopic examination (Figure 2C). Although the throughput of this method is relatively limited, it provides a straightforward route for isolating individual microcolonies at the single-cell level.

A large fraction of natural microbial biodiversity—often referred to as “microbial dark matter”—remains uncultivable under standard laboratory conditions but represents a vast, untapped reservoir of novel secondary metabolites. Exploration of this “dark microbiome” through in situ cultivation has proven to be an effective strategy for discovering unique metabolites, exemplified by the isolation of teixobactin [116]. To increase the throughput of in situ approaches, a microfluidic adaptation known as the microbe domestication pod (MD Pod) was recently introduced [117]. In this system, single cells are encapsulated into agarose microbeads—generated using standard microfluidic protocols—and subsequently incubated within a modified growth chamber (the MD Pod) in their natural environment (Figure 2D). The device was later miniaturized (μMD Pod) to enable its use in small habitats, such as marine invertebrates [118]. The in situ cultivation strategy shows great promise for isolating rare antibiotic producers. A recent study on taxonomic profiling of soil microbiota cultivated in droplets containing soil suspensions enriched with key natural metabolites demonstrated the preservation of low-abundance taxa and recovery of otherwise inaccessible microorganisms [119], emphasizing the critical role of natural environmental factors in microbial cultivability.

**Figure 3:**
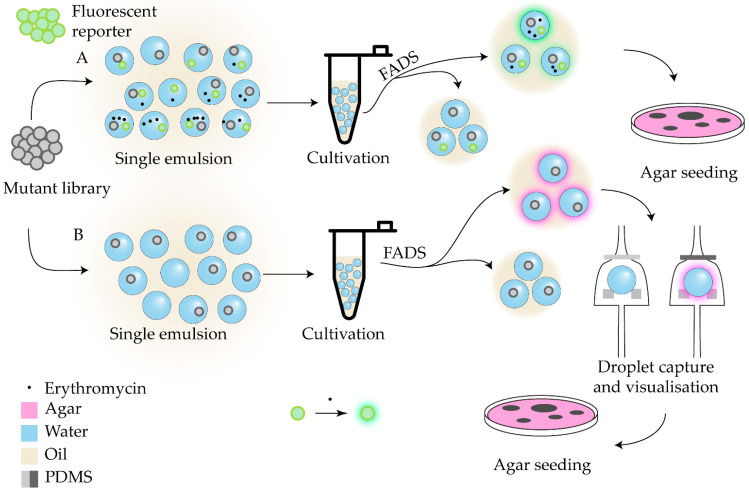
Schematic representation of microfluidic strategies for mutant library screening. (**A**) Emulsion cultivation with FADS. Droplets are generated by flow-focusing. Chips are PDMS replicas of SU-8/silicon master molds fabricated by photolithography and bonded to glass slides. PDMS is surface-modified with Aquapel. (**B**) Emulsion cultivation with on-chip droplet trapping. Droplets are generated by flow-focusing. Chips are PDMS replicas of SU-8/silicon master molds bonded to glass slides. Droplets are immobilized in trapping structures for high-resolution imaging.

A distinct and practically significant challenge in antimicrobial research is the development of industrial microorganisms capable of high antibiotic production. The development of specific antibiotic production biosensors enabled the effective application of previously described droplet-based screening approaches for this task. The integrating of whole-cell biosensors, W/O emulsion, and fluorescence-activated droplet sorting (FADS) led to the establishment of a co-cultivation-based microfluidic screening platform for actinomycetes, termed WELCOME (Whole-cell biosensor and producer co-cultivation-based microfluidic platform for screening actinomycetes) [120]. Subsequent variations in this platform introduced fine-tuning of regulatory element expression through ribosome-binding site engineering of an MphR-based *Escherichia coli* biosensor [121], as well as the development of dual-color biosensors that provide normalized fluorescence outputs [122]. The WELCOME system was successfully applied to select *Saccharopolyspora erythraea* strains hyperproducing erythromycin (Figure 3A).

Screening libraries of chemically induced mutant strains also represents a promising route for diversifying natural metabolite profiles. The metabolic patterns of mutant strains selected using a droplet-based microfluidic platform were shown to be comparable to those obtained via classical screening procedures [123]. In this approach, chemically mutagenized libraries of actinomycetes and bacteria were encapsulated into W/O droplets and processed using an integrated microfluidic device featuring single-droplet trapping sites equipped with pneumatic microvalves. Fluorescence-based detection enabled selective extraction of mutant strains for subsequent mass-spectrometry profiling (Figure 3B).

To summarize, as illustrated in Figure 1 and Figure 2, functional studies of antibiotic producers involve three essential tasks: (1) controlled compartmentalization of microbial community; (2) selective sorting of compartments; (3) specific recovery of target strains. The functional screening of microbial consortia is challenging, as it requires not only sensitive detection and sorting of growth-positive droplets but also recovery of the selected strains for further manipulation.

Droplet-based cultivation provides high generation throughput (Section 2) and effective co-encapsulation with biosensor strains. In terms of generation complexity, single emulsions are more straightforward than double emulsions or hydrogel beads; however, sorting typically requires custom-built droplet sorters, which are expensive and less common than conventional FACS instruments. Overall, both droplet generation and sorting demand specialized equipment and technical expertise, limiting broader adoption. Other intrinsic limitations of droplet cultivation include (1) poor control over leakage of metabolites or biomolecules; (2) restricted exchange of nutrients and gases; (3) limited cultivation duration. Importantly, these features are not unequivocally drawbacks, as the experimental setup can be tuned to exploit them for specific tasks. For example, gas permeability can be adjusted by oil phase variation (mineral vs. fluorinated oil), while reduced gas penetration can be advantageous for anaerobic microorganism isolation. Partial chemical escape from droplets may also enable limited cross-talk without compromising the isolation of individual microenvironments. Previous studies in microbial ecology indicate that droplet isolation enhances the recovery of natural biodiversity [32], suggesting that chemical cross-talk is at least not detrimental to cultivation. Nevertheless, applications to antibiotic producers remain rare [32,42], suggesting that droplet selection and strain recovery pose the main challenge.

Typically, after sorting and de-emulsification, droplet-based functional screens yield bacterial suspensions enriched with active strains for subsequent agar plating and scale-up cultivation. This workflow, however, does not allow for the recovery of individual microcolonies or microbial consortia. The only example of direct droplet content recovery is spatial agar seeding, which is also highly equipment-dependent. In contrast, microwell-array techniques enable straightforward manual isolation of microcolonies but suffer from limited throughput (~10^5^ microwells per chip, whereas droplet incubation approaches can be scaled and typically involve up to 10^7^ individual droplets) and poor reproducibility due to manual handling.

Considering the limitations of current methods, several strategies appear particularly promising for future development.

1. Hydrogel droplets. Although hydrogel bead assays (currently exemplified by MD Pod, Figure 2D) offer lower generation rates, they provide superior gas and nutrient exchange, allowing longer cultivation times. Moreover, agar beads are FACS-compatible and can be processed using standard laboratory equipment. The range of hydrogels suitable for microfluidic applications extends beyond agarose [43], so further application of diverse hydrogel formats could provide more effective droplet-handling approaches.

2. Generation throughput improvement. The rate of droplet generation in microfluidic devices currently limits large-scale emulsion production. Improving generation efficiency could enable direct microbiome analysis without the need for upscaling, which often results in biodiversity loss. A particularly promising strategy is particle template emulsification, initially designed for droplet PCR [124]. This technology limits microfluidic operation to the generation of template nanoparticles, which can then be applied for rapid emulsion generation via vortexing, eliminating the need for continuous-flow microfluidic devices.

3. Alternative sorting approaches. Passive sorting approaches are gaining attention as cost-effective and scalable alternatives to active systems. Droplets can be separated passively on the basis of deformability [125], interfacial tension [126], size [127] and other physical parameters [42]. The development of passive sorting techniques for antibiotic producer screening could have a transformative impact, overcoming one of the major barriers to widespread application—the high cost and limited accessibility of active droplet-sorting equipment.

## 4. Antibiotics: Screening and Mechanism Elucidation

**Figure 4:**
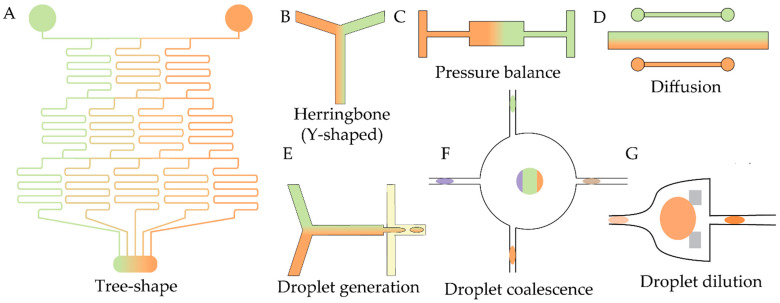
Gradient generators for microfluidic applications. (**A**–**D**) Examples of on-chip gradient generation architectures. (**E**–**G**) Strategies for creating controlled concentration gradients within droplet populations.

After hit identification, the next step in drug development is a detailed analysis of antimicrobial activity and mode of action (MoA). Microfluidic technologies enable substantial reductions in sample consumption, support high-throughput combinatorial screening, and provide time-resolved, single-cell-level insights into drug-induced stress responses. Recent applications are summarized in this section. For both MoA studies and alternative strategies for determining inhibitory concentrations, a critical requirement is the creation of controlled gradients of one or more components. Accordingly, and in addition to the chip-fabrication techniques described in Section 2, we first briefly summarize the established technical approaches to gradient generation. Most of the existing solutions are based on either on-chip fluid manipulation (Figure 4A–D) or generation, merging, and diluting droplets (Figure 4E,F) [128].

It is worth noting that microfluidic technologies are constantly evolving, including the development of new approaches to creating gradients of chemical compounds. For example, to test combinations of anticancer drugs on single cells, an alternative tree-shaped gradient generation architecture was developed [129]. Another recently designed compound mixer is a 3D microchannel network design for multiple concentration gradient formations and mixed solution combinations [130]. The development of novel systems for clinical AST includes the introduction of novel on-chip (e.g., MVM^2^ platform [131]) and off-chip (e.g., microfluidic mixer [132]) gradient generators. In this rapidly changing field, transferring technologies and approaches from related fields holds promise for effective development of novel platforms; recently, cross-disciplinary gradient devices were comprehensively summarized [133].

### 4.1. Advancements in Screening Approaches

Screening natural or synthetic compounds for potential drug candidates remains significantly constrained by the low throughput and labor-intensive operations required for bioactivity testing. Microfluidic technologies have emerged as powerful tools for analyzing the microbial susceptibility to diverse agents and their combinations. The most rapidly advancing area within this field is AST, aimed at facilitating rapid therapeutic decision-making in clinical settings. These methods are designed for the fast evaluation of clinical isolates resistance to antibiotics, requiring minimal instrumentation and providing short turnaround times. While numerous recent reviews summarize microfluidic AST approaches: single-cell AST [69]; alternative AST technologies [70,74]; technical innovations and therapeutic applications [75]; DNA amplification-based [71] and other genotyping AST approaches [82]; antimicrobial-resistance related AST [72,73,79], including multidrug resistance [81]; AST-based disease diagnostics [76,77,78]; color-based microfluidic AST [80]; etc. The present section focuses on the methods developed for (or adapted to) in vitro drug discovery and combinatorial drug development.

#### 4.1.1. Screening of Chemicals and Their Combinations

Analysis of the latest literature on microfluidic approaches to drug screening shows that the majority of applied methodologies are droplet-based. The first challenge in such workflows is the controlled generation of multicomponent droplets. Once droplets containing bacteria and defined drug concentrations are produced, bacterial growth in each droplet must be assessed in relation to the corresponding antibiotic dose. The solutions developed for these tasks are tightly interconnected: the chosen strategy for droplet content multiplexing largely determines the applicable detection methods. This intrinsic relationship allowed us to group droplet-based approaches into two distinct categories: (1) imaging-based methods and (2) spatially controlled techniques.

**Figure 5:**
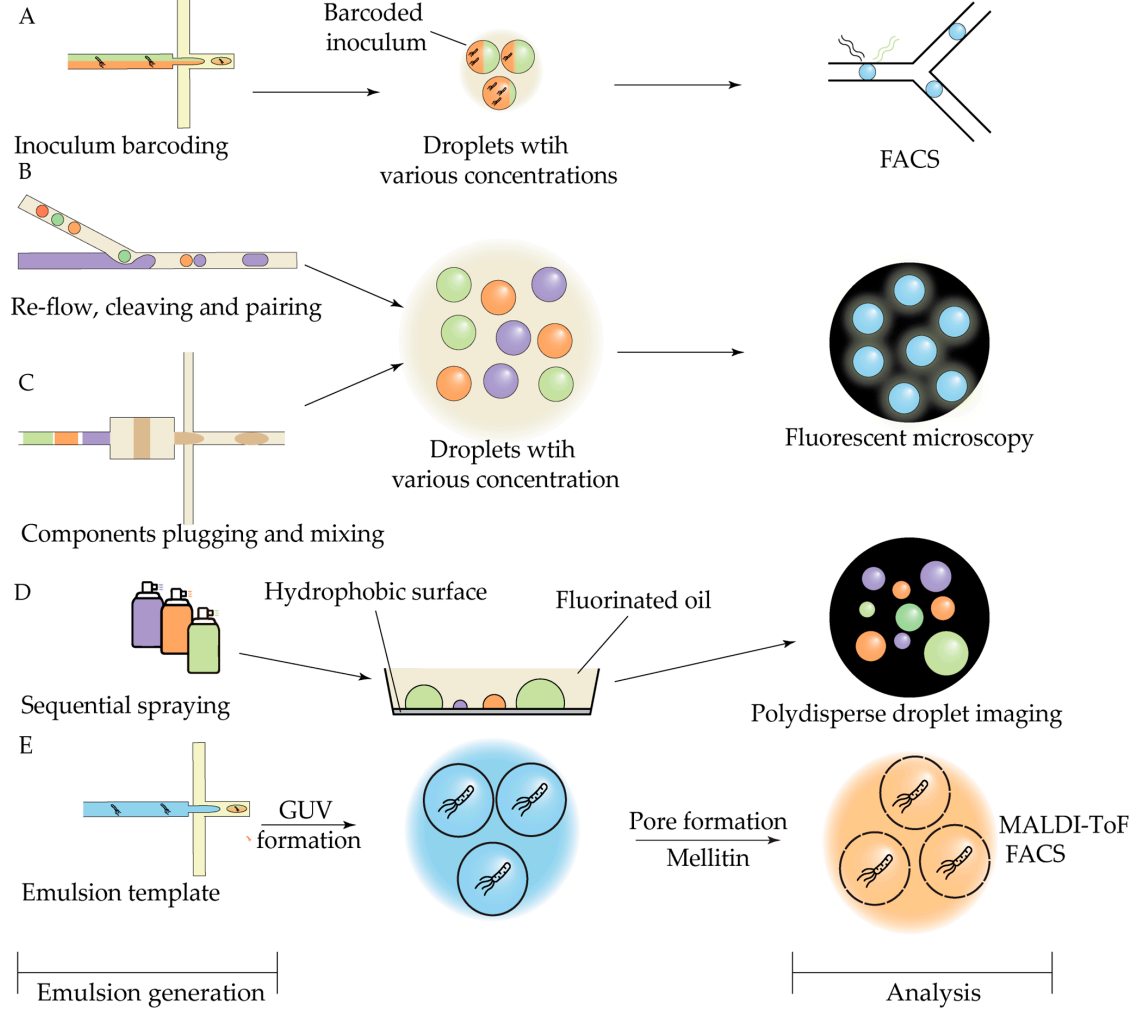
Droplet-based screening approaches with imaging-based concentration control. (**A**) Inoculum barcoding in emulsions. Droplets are generated by flow-focusing, and bacterial inoculum is barcoded with fluorescent dyes. Analysis and sorting are performed by FACS. (**B**) Reflow-based droplet multiplexing. Droplets are generated using a T-junction. Chips are multilayer PDMS replicas of SU-8/silicon master molds, bonded together. Droplets are reflowed through the chip for multiplexed reagent addition and analyzed by fluorescence imaging. (**C**) Pulse-mixing of sample plugs for combinatorial droplet generation. Droplets are generated by flow-focusing. Chips are PDMS replicas of SU-8/silicon master molds bonded to glass slides. Concentrations are decoded and analyzed by fluorescence imaging. (**D**) Sequential spraying for droplet generation. Droplets are formed by sequential spraying of fluorophore-encoded components onto a hydrophobic surface and subsequently analyzed by fluorescence imaging. (**E**) Giant unilamellar vesicle (GUV) encapsulation strategy. Template droplets are generated by flow-focusing using the chip, fabricated by DRIE in silicon, and bonded to a glass substrate with micromachined access holes. GUVs are analyzed by FACS or MALDI-TOF.

The first approach to control droplet interior is co-encapsulation with an array of fluorescent dyes. Fluorescent droplet barcoding is typically combined with off-chip bulk incubation, followed by aliquot sampling and microscopy imaging [134,135,136] (Figure 5). A representative example is the use of color-coded control of cell density to improve the accuracy of inoculum-effect evaluation [137] (Figure 5A).

Effective screening of single drugs or drug combinations in droplets requires more sophisticated assay designs, typically involving gradient generation via precise droplet manipulation. The most straightforward strategy is to generate separate droplet populations, each containing a distinct antibiotic concentration [138], but this approach is inherently limited in throughput. Several gradient generation methods have been outlined above (Figure 4), and recent studies have adapted or refined these droplet-control strategies specifically towards screening applications.

One example is a novel integrated droplet-merging chip that enables re-flow of pre-formed droplets and synchronized reagent addition via droplet cleaving, thereby providing passive control over droplet composition (Figure 4B) [134]. Another innovative approach achieves droplet multiplexing and tracking via combinatorial sample preparation [135]: the droplet generator is supplied with automatically dosed and merged sample plugs, each encoded with fluorescent dyes, with merging driven by oscillatory flow (Figure 5C). These designs considerably simplify multiplexing at the droplet level but still require relatively sophisticated equipment.

A conceptually different method eliminates microfluidic devices altogether. Here, droplets are generated by sequential spraying of fluorophore-encoded components onto a hydrophobic surface (Figure 5D). Although this device-free approach yields polydisperse droplets, fluorescence staining allows quantitative determination of compound concentrations by imaging [136]. However, image analysis becomes substantially more complex and error-prone, since the non-uniform geometry of each droplet must be determined individually.

A fundamental challenge of fluorescent barcoding lies in the differing physicochemical properties of the dyes and the tested compounds, which can cause variable leakage and inconsistencies in the measured results [139]. It is worth noting that all droplet-based assays can suffer from chemical escape from droplets. Limited cross-talk may be advantageous in microbiome cultivation studies, allowing inter-droplet chemical signaling within isolated microenvironments. In susceptibility assays, by contrast, maintaining consistent antibiotic and dye concentrations across droplets is critical. This limitation should be taken into account in the design of droplet experiments, for example, by means of the prediction of antibiotic retention in emulsions [139]. A recent alternative approach addresses permeation control by using double-emulsion-templated giant unilamellar vesicles (GUVs) as microincubators for bacterial cultivation [140]. In this system, the permeability of the polydimethylsiloxane (PDMS)-based block copolymer membrane is controlled by introducing biopores formed by the amphiphilic peptide melittin (Figure 5E). Despite selective permeability, the application of core–shell beads suffers from a more difficult and slower generation than droplets. Moreover, the current design is not compatible with gradient generation; nonetheless, this GUV-based approach may be the foundation of future screening platforms.

**Figure 6:**
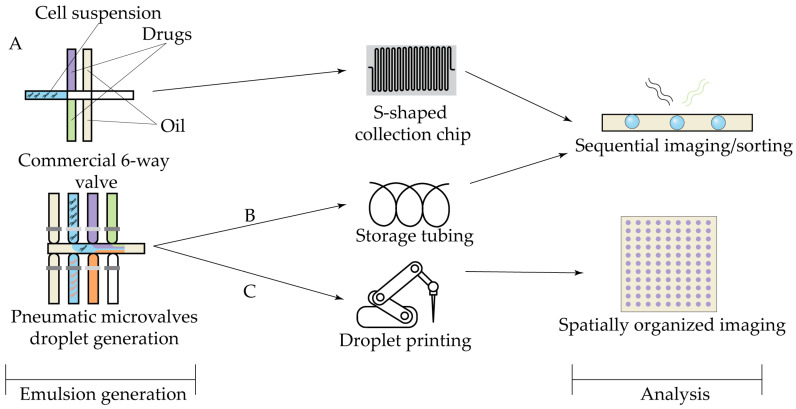
Microfluidic screening approaches with spatially controlled droplets. (**A**) Combinatorial screening with spatially ordered droplets. Emulsions are generated using a commercial 6-way junction and a T-junction geometry. The storage chip is a PDMS replica of a micromachined aluminum master mold. Droplets are analyzed by fluorescence imaging. (**B**) Pneumatically controlled sequential combinatorial screening. Droplets are generated by a T-junction. Chips are PDMS replicas of SU-8/silicon master molds fabricated by photolithography. Droplets are analyzed by fluorescence imaging. (**C**) RoboDrop platform. Droplets are generated by a T-junction in PDMS chips replicated from SU-8/silicon master molds. After generation, droplets are robotically printed onto arrays and imaged using a commercial camera.

The second group of methods of droplet interior control relies on spatially encoded reagent gradients, enabled by droplet generation via programmable pneumatic microvalves (Figure 6). In these systems, automated droplet production is typically followed by ordered transfer into tubing (Figure 6B) or an S-shaped collection chip (Figure 6A) for incubation and subsequent ejection through the detection channel for sequential detection. By design, these approaches circumvent the dye retention limitations of fluorescent barcoding and have been successfully applied to combinatorial antibiotic screening [141,142].

Microvalve-based droplet systems, however, remain limited by the complexity of microdevice design and fabrication. Nonetheless, related technologies continue to advance toward greater precision and operational simplicity. The recently developed Tubing-Eliminated Sample Loading Interface (TESLI) utilizes vacuum-based actuation to streamline sample handling [143]. Another notable innovation, the Artificial Intelligence-accelerated High-Throughput Combinatorial Drug Evaluation System (AI-HTCDES), achieves effective combinatorial dosing using readily available commercial multiway valves and programmable syringe pumps (Figure 6A) [141], addressing several limitations inherent to custom-fabricated devices.

The imaging of spatially ordered droplets generates large datasets that demand automated analysis. To address this challenge, deep learning-based image processing was incorporated into the AI-HTCDES platform [141]. In this assay, sequential bright-field imaging of over 7000 droplets was efficiently analyzed using machine learning algorithms to identify bacterial growth automatically. Another problem of sequential detection is throughput and scalability, so the detection approach was recently upgraded in the Robotic-Printed Combinatorial Droplet (RoboDrop) [144] system by the addition of a robotic arm after tubing, which automatically prints the ejected droplets onto an array, enabling simultaneous imaging of thousands of droplets using a standard camera (Figure 6C).

In summary, widespread adoption of high-throughput droplet screening methods remains limited by the need for specialized equipment and custom-built microfluidic platforms. Nevertheless, the field is trending toward greater democratization, as demonstrated by devices constructed from commercially available components and by automation that reduces user expertise requirements. Integrating these advancements into standardized, modular platforms, supported by robust and accessible data analysis pipelines, will be essential for enabling broad implementation of microfluidic high-throughput screening technologies.

Bioactivity analysis of natural compounds, complex mixtures and fractions is a distinct task that requires a different strategy than the combinatorial screening approaches discussed above. The straightforward observation of growth inhibition in conventional microfluidic channels has proven unsuitable for detecting antimicrobial compounds within complex natural product matrices [145]. An intriguing recent innovation to overcome this problem has adapted the SlipChip droplet generation strategy towards screening natural products with antimicrobial activity in a nanoliter matrix SlipChip (nm-SlipChip) [146]. SlipChip devices present an attractive alternative to standard microfluidic protocols, offering comparable precision in fluid manipulation without the need for external instrumentation. A detailed review of SlipChip design principles and their applications in digital bioassays was recently published [147].

**Figure 7:**
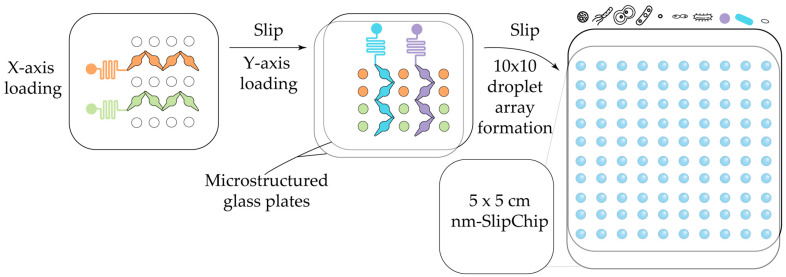
Schematic representation of nm-SlipChip. Chips are fabricated by selective glass etching.

In SlipChip, droplets are generated through the relative movement of two closely aligned microstructured glass plates, each patterned with microwells and microducts on their contacting surfaces. In an nm-SlipChip assay, ten antimicrobial candidates (either pure compounds or extract fractions) are simultaneously tested against ten bacterial cultures within microwell-generated droplets (Figure 7). The compounds or fractions (and potentially different drug concentrations) are loaded into the *X*-axis channels of the chip, while bacterial suspensions are introduced into the *Y*-axis channels. A controlled sliding motion merges the two arrays, initiating on-chip incubation. Following incubation, droplet growth inhibition is assessed microscopically. Compared with classical bioactivity testing methods, this approach not only minimizes sample consumption but also reduces analysis time to approximately three hours. Aside from the fabrication of the two glass plates comprising the nm-SlipChip by etching, this approach does not require any specialized equipment. Importantly, it integrates droplet generation and spatial control of droplet contents within a single device, and it is compatible with the recovery of droplets of interest for downstream analysis or alternative detection methods. Despite offering dramatically lower throughput compared with off-chip droplet generation (100 droplets per chip versus ~10^5^ droplets in a typical droplet experiment), this approach remains promising for specialized applications in drug development, particularly for testing HPLC fractions, because it enables a highly miniaturized assay with low sample consumption.

#### 4.1.2. Screening of DNA-Encoded Antimicrobials

**Figure 8:**
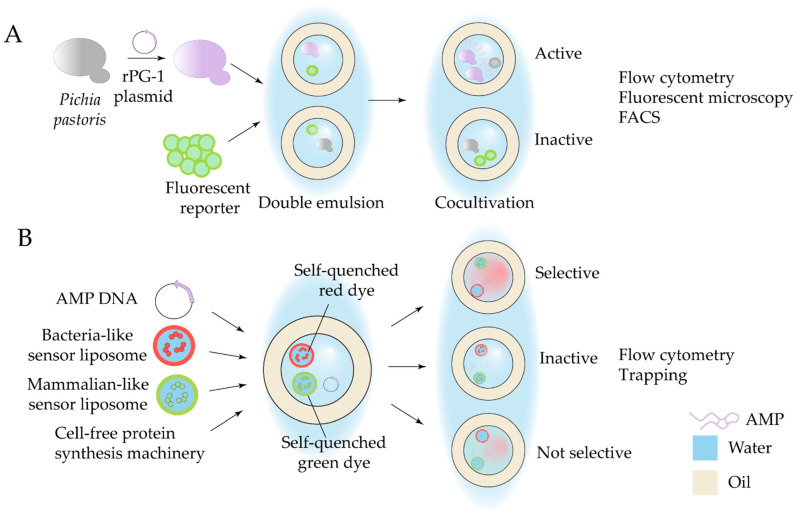
Approaches to peptide library screening. (**A**) Co-cultivation in double emulsions. Droplets are generated by flow-focusing. Chips are PDMS replicas of SU-8/silicon master molds fabricated by photolithography and bonded to glass slides. PDMS surfaces are modified with PVA, Mowiol, trichloro(octadecyl)silane, or Aquapel. (**B**) Multiplexed cell-free peptide screening. Chips are PDMS replicas of SU-8/silicon master molds, with two bonded PDMS layers.

Specialized microfluidic screening strategies can be applied to evaluate libraries of DNA-encoded antimicrobial peptides (AMPs). Genetically encoded sequences of AMPs enable the generation of libraries containing unnatural modifications through directed evolution techniques [148]. AMP libraries can be produced either by chemical synthesis, heterologous recombinant expression systems, or cell-free translation platforms. In all cases, high-throughput screening of target biological activities is essential for hit identification, making microfluidic methods particularly suitable for this area of research. For example, droplet-based screening in a double W/O/W emulsion was applied for the evaluation of protegrin-1 production in *Pichia pastoris* yeast, establishing a platform for screening of recombinant AMP activity against Gram-negative bacteria [149] (Figure 8A).

Since most AMPs act through membrane permeabilization and frequently lack selectivity, often displaying high hemolytic activity, strategies that assess their membrane specificity are critical. To address this need, a microfluidic system enabling simultaneous cell-free production and screening of AMP membrane specificity was developed [150]. In this approach, AMPs are produced within W/O/W double emulsion droplets, co-encapsulated with large unilamellar sensor vesicles, that generate a membrane-specific fluorescence signal. Two distinct populations of sensor liposomes—with bacterial-like or mammalian-like lipid compositions—are preloaded with self-quenched fluorescent dyes. Dye leakage in droplets can be observed by droplet trapping of FACS and is proportional to the preference of the AMP for the specific membrane composition (Figure 8B). The sensor liposome-based approach was further shown to be applicable to detecting and evaluating membrane-active antimicrobial peptides in non-ribosomal peptide synthase mutant libraries, exemplified by heterologous production of gramicidin S in *E. coli* [151].

An interesting alternative approach allows for the assessment of AMP selectivity towards whole cells based on microfluidic impedance cytometry [152]. In this approach, bacterial cells and human red blood cells are treated with AMP off-chip, and the resulting individual or mixed samples are analyzed using an impedance cytometer. The system records electrical signatures of single cells, reflecting AMP-induced changes in membrane integrity. The key advantage of this approach lies in its rapid, label-free, and co-culture-compatible evaluation of AMP selectivity, providing physiologically relevant insights into peptide activity.

#### 4.1.3. Bacteriophage Evaluation and Development

Bacteriophages are increasingly recognized as a promising alternative strategy for combating antibiotic-resistant bacteria. However, conventional phage isolation methods remain labor-intensive and may introduce significant bias [153]. Microfluidic technologies offer new opportunities for phage research by providing precise control over environmental parameters and enabling high-throughput experimentation. Despite this potential, reported applications of microfluidics in phage biology are still relatively limited.

**Figure 9:**
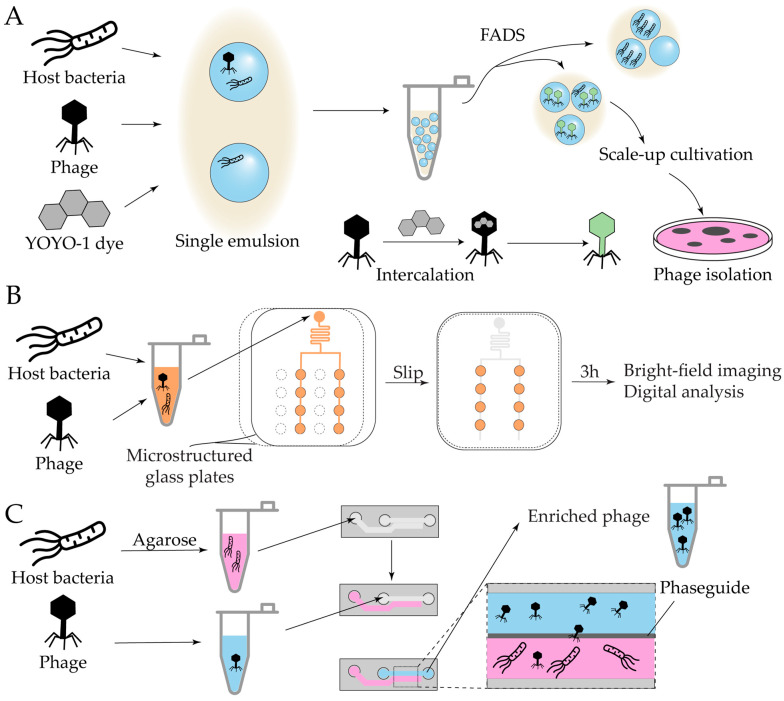
Microfluidic-based phage discovery pipelines. (**A**) Fluorescence-assisted isolation of phages in single emulsions. Droplets are generated by a commercial microfluidic chip. Positive droplets are selected with FADS. (**B**) SlipChip-based digital phage analysis. The device consists of two glass plates fabricated by selective etching. Phage-induced host cell lysis is monitored by bright-field microscopy. (**C**) On-chip phage isolation using a commercial microfluidic platform.

The first demonstration of a droplet-based approach for isolating infectious phages involved the co-encapsulation of phages and *E. coli* host cells in W/O droplets [154]. The fluorescent nucleic acid dye YOYO-1 was used to label phage DNA; due to its cell-impermeant nature, it selectively stained viral particles without affecting host cells. Droplets were analyzed and sorted using an On-chip Droplet Selector, allowing for the isolation of target droplets into individual wells of a 96-well plate (Figure 9A). This proof-of-concept study established the feasibility of droplet microfluidics for phage discovery and has since inspired further developments, as exemplified by two recent preprints expanding this approach [155,156].

The aforementioned technology for on-chip droplet generation was also adapted towards phage quantification in digital phage SlipChip (dp-SlipChip), relying on bright-field microscopy for host bacterial cell growth detection [157] (Figure 9B). An alternative on-chip cultivation strategy utilized a commercially available microfluidic platform (Mimetas Organoplates), originally developed for eukaryotic cell cultures, and repurposed it towards phage isolation and quantification [158]. Each chip consists of two parallel microchannels converging into a shared interaction chamber separated by a narrow phaseguide. In this setup, one channel is filled with bacteria immobilized in agarose gel, while the adjacent channel contains phage suspensions in liquid medium (Figure 9C). Incubation yields phage enrichment.

#### 4.1.4. Antibiofilm Activity Testing

Biofilm development plays a pivotal role in microbial resistance to treatment in the clinic. Consequently, compounds that can either prevent biofilm formation or disrupt mature biofilms are of significant therapeutic interest. Microfluidic platforms have proven particularly valuable in biofilm research, as they provide precisely controlled, dynamic environments that closely mimic natural growth conditions.

**Figure 10:**
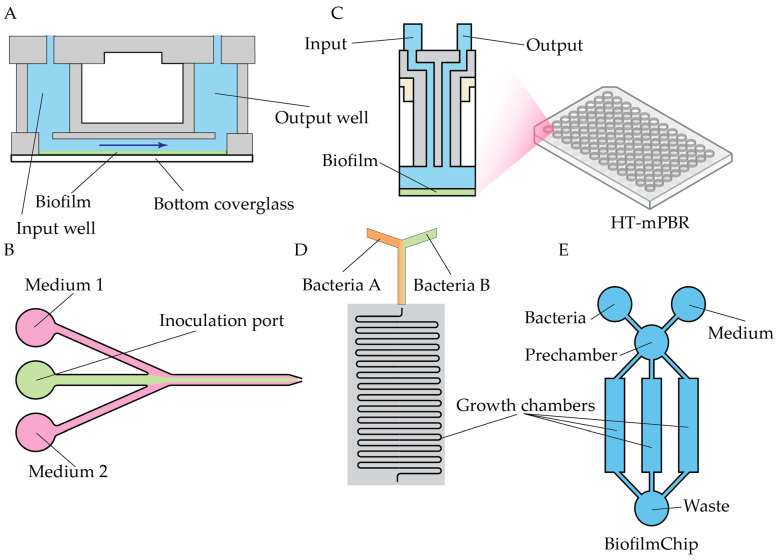
Examples of microfluidic biofilm models for activity screening. (**A**) BioFlux system. The chip is a PDMS replica of an etched silicon wafer. (**B**) Flow-focused biofilm formation model. Chips are PDMS replicas of SU-8/silicon master molds fabricated by photolithography and bonded to glass slides. (**C**) High-throughput microfluidic perfusion biofilm reactor (HT-μPBR). The device is assembled from multiple components, combining micromachined parts with PDMS replicas. (**D**) Modular platform for biofilm drug screening. Chips are PDMS replicas of SU-2050 master molds fabricated by photolithography (**E**) BiofilmChip platform. Chips are PDMS replicas of Ordyl/glass master molds fabricated by lithography.

Well-established open biofilm models are available commercially, e.g., BioFlux [159], and similar microfluidic chambers remain widely used for biofilm studies and screening applications. In these devices, biofilms are formed within a microchannel attached to a glass slide under continuous flow conditions (Figure 10A). Even this relatively simple experimental configuration yields substantially more physiologically relevant data than traditional static models. For instance, the BioFlux system enabled the identification of antibiofilm activity of the peptide Temporin L against *P. fluorescens*, demonstrating effects beyond its known antimicrobial properties [160]. A similar setup was employed to reveal the antibiofilm efficacy of murepavadin against *P. aeruginosa* [161] and to study the modulation of intestinal bacterial adhesion by naringenin derivatives [162].

Further applications of BioFlux-based models include investigations of ultrashort antifungal lipopeptides against *Candida* biofilms [163], and marine invertebrate-derived AMPs active against opportunistic pathogens, such as *Aurelia aurita* and *Mnemiopsis leidyi* [164]. These studies demonstrated that microfluidic biofilm systems can generate reproducible and biologically meaningful results relevant to complex natural and clinical environments.

A three-port modification of the BioFlux design was later introduced, allowing the application of spatially separated flows of distinct media within a single device. This configuration was validated for antibiofilm screening [165] (Figure 10B). To further improve screening capacity, a high-throughput microfluidic perfusion biofilm reactor (HT-μPBR) was recently developed specifically for antibiofilm testing [166] (Figure 10C). Compared with the standard BioFlux system, HT-μPBR offers enhanced shear control and full compatibility with standard microtiter plates and incubator setups.

Research on the formation and eradication of mixed-species biofilms is critically important, as many infections in the human host are caused by polymicrobial biofilms. A BioFlux device was inoculated with a mixed *S. aureus* and *Pseudomonas aeruginosa* culture to create a model of the diabetic foot environment for antibiotic screening [167]. A more advanced platform incorporating a herringbone micromixer for combining separate microbial flows, coupled with optofluidic imaging for real-time monitoring, was applied to study mixed *S. aureus + C. albicans* biofilms [168] (Figure 10D). Recently, an even more sophisticated system for modeling biofilms—including polymicrobial and clinically derived communities—was introduced [169]. The BiofilmChip design (Figure 10E) features a prechamber and three growth chambers with optimized geometry, supporting both confocal microscopy and impedance measurements. Validation using clinical samples demonstrated that BiofilmChip effectively supports the growth and characterization of polymicrobial biofilms.

Bacterial colonization on solid surfaces and subsequent biofilm formation pose particular challenges in the context of implanted device application. Antimicrobial coating and materials development is therefore an active research area, though their reliable screening and evaluation remain difficult. Microfluidic technologies have provided efficient solutions to this problem. For instance, a custom microfluidic device was used to investigate the attachment behavior of *P. aeruginosa* and *S. aureus* species on a nanostructured surface under fluid wall shear stress [170]. Fluorescent staining and microscopic visualization enabled detailed quantification of bacterial adhesion. Another study employed a standard biofilm model to assess the antimicrobial performance of AMP-functionalized surfaces [171]. In this work, continuous monitoring of *E. coli* growth revealed crucial factors to aid AMP-grafted coating design.

To conclude, despite their clear advantages over static assays, current microfluidic antibiofilm platforms also have important limitations. BioFlux and similar open-channel systems, while relatively user-friendly, offer only coarse control over shear profiles and often suffer from channel clogging and poorly standardized inoculation procedures, which impair reproducibility. More advanced devices, such as HT-μPBR and BiofilmChip, provide better control of flow and multiplexing, but this comes at the cost of increased fabrication complexity and higher equipment demands. Many platforms rely on PDMS, raising concerns about the adsorption of antibiotics and signaling molecules, as well as evaporation during long-term experiments. Mixed-species and biomaterial-oriented models frequently face challenges in reproducible seeding, long-term stability, and efficient biomass recovery for downstream analysis. It is important to note that the throughput of microfluidic biofilm models is intrinsically constrained by their on-chip experimental design. Even well-plate-compatible formats typically allow for the analysis of no more than 96 conditions per run, while most custom microfluidic chips contain far fewer growth chambers, further limiting experimental throughput. Finally, the large imaging datasets generated by these systems are not yet matched by standardized analysis pipelines, which limits interoperability and broader adoption. Together, these constraints highlight the need for more robust, standardized, and material-aware designs if microfluidic antibiofilm assays are to transition to broadly applicable platforms for drug and materials development.

### 4.2. Studies on Antibiotic Mode of Action

Identification of a hit compound or effective drug combination represents only the first stage in antimicrobial drug development. The next critical step is elucidating the molecular mode of action (MoA) underlying the observed biological effects. In contrast to screening-oriented platforms, microfluidic systems designed for mechanistic studies typically shift from endpoint growth detection toward time-resolved single-cell imaging, requiring modified experimental setups and device architectures.

#### 4.2.1. Single-Cell Phenotyping for MoA Studies

**Figure 11:**
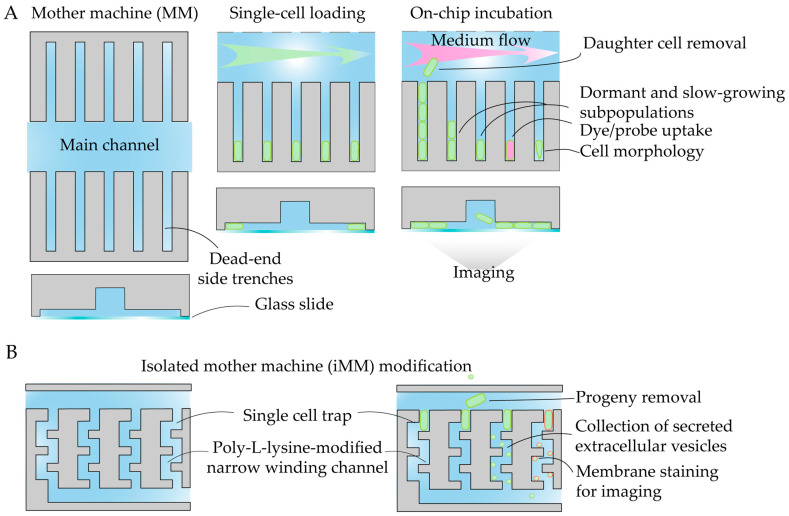
Schematic representation of (**A**) the mother machine (MM) and (**B**) the isolated mother machine (iMM). Both devices are fabricated as PDMS replicas of SU-8/silicon master molds produced by photolithography and bonded to glass slides.

One of the most established microfluidic designs for single cells is a configuration nicknamed the “mother machine” (MM, [172]). The MM consists of a large central flow channel intersected by numerous perpendicular, single-cell-wide dead-end side trenches. During inoculation, individual bacterial cells become trapped in these narrow side channels, while their progeny are continuously flushed away by a constant flow of fresh medium in the main channel (Figure 11A). Time-lapse microscopy in this configuration enables the observation of single-cell lineages over hundreds of generations [173]. The MM remains widely applied in recent antibiotic MoA studies (Table 2).

Building upon this principle, an innovative design termed the isolated mother machine (iMM) was developed to capture extracellular vesicles (EVs) [174]. In this system, individual cells are retained in narrow side channels similar to the MM, but all daughter cells are immediately removed by continuous flow. The winding microchannels are selectively coated with poly-l-lysine, forming a positively charged surface that enables electrostatic trapping of negatively charged EVs (Figure 11B). Trapped EVs can be either visualized and enumerated or recovered for further analysis.

**Figure 12:**
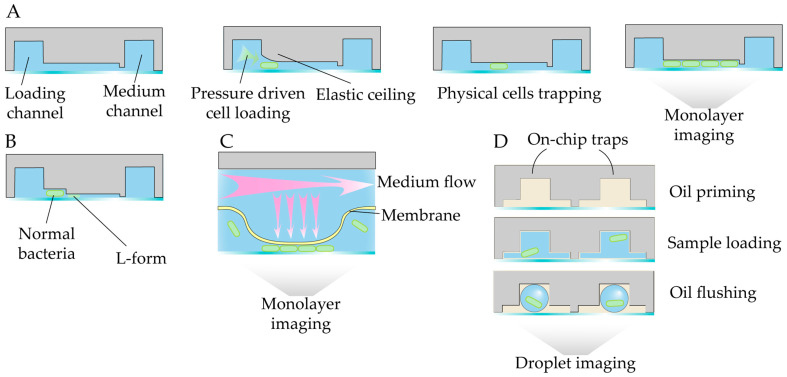
Microfluidic experimental setups for on-chip cell imaging. (**A**) Principle of monolayer trap operation. Monolayer traps designed for L-froms (**B**) and for mycobacteria (**C**). (**D**) Operating scheme of on-chip droplet generation. The devices are fabricated as PDMS replicas of SU-8/silicon master molds produced by photolithography and bonded to glass slides.

Another widely adopted strategy for single-cell imaging involves trapping bacteria in monolayers to track microcolony dynamics. Various devices, including commercially available ones (e.g., CellASIC^®^ ONIX B04A-03 bacterial plates), have been employed for the detailed investigation of bacteria–drug interactions (Table 2). Their working principle consists of loading and trapping of single cells in a one-cell-high monolayer with subsequent time-resolved imaging of microcolony development (Figure 12A). A device with variable chamber height has been applied to selectively trap normal bacterial cells or cell-wall-deficient L-forms (Figure 12B) [175]. An alternative design employs hydropneumatic chambers to form bacterial monolayers (Figure 12C). Such systems have been utilized in studies of *Mycobacterium smegmatis*, including the μDeSCRiPTor platform, which is a microfluidic platform for dynamic single-cell pheno-tuning compound screening, used for mechanism-informed development of anti-tubercular adjuvant therapies [176].

A different mechanistic approach involves on-chip generation of immobilized droplets, enabling the real-time observation of bacterial growth and lysis dynamics (Figure 12D). This setup has been applied to study *E. coli* challenged with phages [177] and antibiotics [178]. Immobilized droplet arrays provide stable and isolated microenvironments for time-resolved imaging of individual bacterial responses.

**Table 2:** Summary of single-cell imaging-based studies of modes of antimicrobial action.

Compound	Label	Device	Detection Method	Test Strain	Observed Effect	Ref
Ultrashort peptides	Live/Dead stain	Figure 11A MM	Fluorescent imaging	*E. coli*	Variable single-cell killing kinetics	[179]
Roxithromycin	Labeled antibiotic	Figure 11A MM	Fluorescent imaging	*E. coli*, expressing phage secretin f1pIV	Drug uptake	[180]
Ofloxacin	Natural antibiotic fluorescence	Figure 11A MM	Epifluorescent imaging	*E. coli*	Drug uptake	[181]
Polymyxin	GFP, membrane stain	Figure 11B iMM	Fluorescent imaging	*E. coli*	Extracellular vesicle secretion	[174]
protein Ψ-capsids	-	Figure 11A MM	Bright-field microscopy	*E. coli*	Single-cell killing kinetics	[182]
Proof-of-concept study	Labeled vancomycin	Figure 11A MM	Fluorescent imaging	*E. coli*	Outer membrane damage by probe uptake	[183]
Polymixin	-	Figure 12B	Bright-field microscopy	*E. coli*	L-form morphology and proliferation	[175]
Aminoglycosides and fluoroquinolones	GFP, mCherry	Figure 12A	Fluorescent imaging	*E. coli* with genetic compartment markers	Hyperosmotic shock, cytoplasmic condensate ion	[184]
Meropenem, berberine	Live/Dead stain	Figure 12A	Bright-field and fluorescent microscopy	*A. baumannii*	Single-cell growth kinetics under combination therapy	[185]
Listeriolysin S	GFP, Sytox blue	Figure 12A	Fluorescent imaging	*Listeria monocytogenes*	Contact-killing by producing strain	[186]
Moxifloxacin	GFP, PI	Figure 12C	Bright-field microscopy	*M. smegmatis*	Single-cell dose response	[187]
M06 pheno-tuning compound	mCherry, GFP	Figure 12C	Fluorescent imaging	*M. smegmatis*	Single-cell phenotypes	[176]
Phages	GFP	Figure 12D	Fluorescent imaging	*E. coli*	Growth and lysis kinetics	[177]
Ciprofloxacin	mRFP	Figure 12D	Fluorescent imaging	*E. coli*	Growth kinetics	[178]

To summarize, most recent studies on antibiotic mode of action have been carried out using well-established microfluidic platforms, primarily mother machine-type devices and monolayer traps. In each case, however, the experimental design is carefully adapted to the specific biological question. Despite their clear advantages, microfluidic conditions can introduce additional biases, including altered growth dynamics under shear stress, uncontrolled absorption of dyes, antibiotics, or probes into PDMS, and imaging-related artifacts. Consequently, key findings obtained in microfluidic systems should be validated using a set of orthogonal, complementary methods.

#### 4.2.2. Membrane Interaction Modeling

Understanding the interaction of antibiotics with biological membranes is essential for elucidating their molecular mode of action, particularly for compounds whose activity depends on membrane permeation, disruption, or transport. Microfluidic platforms provide a unique opportunity to model these interactions under well-controlled, physiologically relevant conditions. Recent advancements in this area have produced versatile tools for dissecting drug–membrane interactions, several of which are highlighted below.

**Figure 13:**
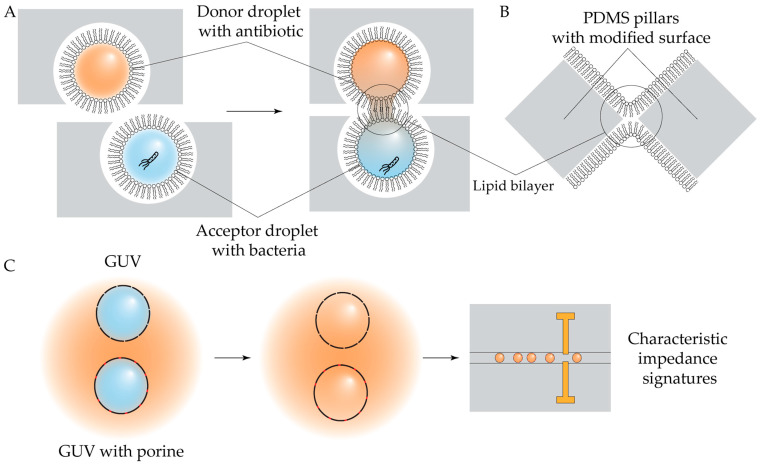
Designs specialized towards mechanistic studies. (**A**) Droplet model of the interface bilayer. The chip is fabricated by laser cutting of PMMA sheets, and drug transport is monitored by fluorescence imaging. (**B**) Artificial planar cell membrane platform. The chip is produced by PDMS replication of a silicon master patterned by DRIE. The PDMS surface is modified with trichloroperfluorooctylsilane (**C**).

To emulate drug bioavailability in intracellular infections, a specialized model based on droplet interface bilayers (DIBs) was developed [188]. In such infections, the host–cell membrane forms a barrier between the pathogen and antimicrobial agents. The DIB model reconstitutes this condition by forming lipid bilayers at the interfaces of lipid-coated water-in-oil (W/O) droplets. Arrays of droplets are generated on a chip equipped with a movable chamber, following previously established methods [189]. Controlled contact between antibiotic-containing droplets and bacteria-loaded droplets allows for the monitoring of drug diffusion across the DIB, mimicking intracellular drug exposure (Figure 13A).

Another approach to the study of drug–membrane interactions utilizes freestanding planar lipid bilayers, though their formation and buffer exchange have traditionally been difficult to control. A recently introduced on-chip platform allows arrayed planar bilayer formation and was successfully applied towards the study of the effects of azithromycin on the mechanical properties of POPC lipid bilayers (Figure 13B) [190].

Model membrane permeabilization under antibiotic treatment can also be examined using GUVs. A label-free, impedance-based on-chip GUV detection system integrating microelectrodes within the microfluidic channel was developed and validated using norfloxacin as a model compound [191] (Figure 13C). Notably, this system could distinguish between antibiotic permeation through the lipid bilayer and transport mediated by inserted porin channels, offering detailed mechanistic insights into drug–membrane interactions.

Taken together, these membrane-mimetic microfluidic platforms allow for observations that bridge simplified biophysical systems with the complex environments encountered during intracellular and extracellular infections. Despite their current specialization, continued integration of these tools with microfluidic handling, automated analysis, and high-throughput assay formats is likely to expand their applicability in membrane targeting studies.

## 5. Pathogens: Stress Responses and Resistance Development

Systematic studies of how bacterial populations respond to antibiotic stress and how resistant subpopulations emerge provide the mechanistic and quantitative foundation for rational antimicrobial therapy. Microfluidic technologies have become indispensable in this context, offering precise environmental control, high temporal resolution, and single-cell-level insight into the complex dynamics of resistance acquisition. This section summarizes recent microfluidic studies that illuminate the diverse pathways through which pathogens survive antimicrobial treatment.

### 5.1. Genetic Resistance

Adaptive laboratory evolution (ALE) is a key method for investigating the emergence of antibiotic resistance and elucidating its underlying mechanisms. Microfluidic systems have transformed ALE workflows by accelerating mutant generation, improving environmental control, and enabling high-throughput selection of desired traits [54]. Beyond mechanistic insights, these approaches facilitate detailed preclinical resistance prediction, potentially extending the clinical lifespan of new antimicrobial agents [53]. A recent review summarizes current approaches to the assessment of resistance development, focusing on technologies of laboratory evolution on resistance mutants at the hit-to-lead stage of drug development [53].

**Figure 14:**
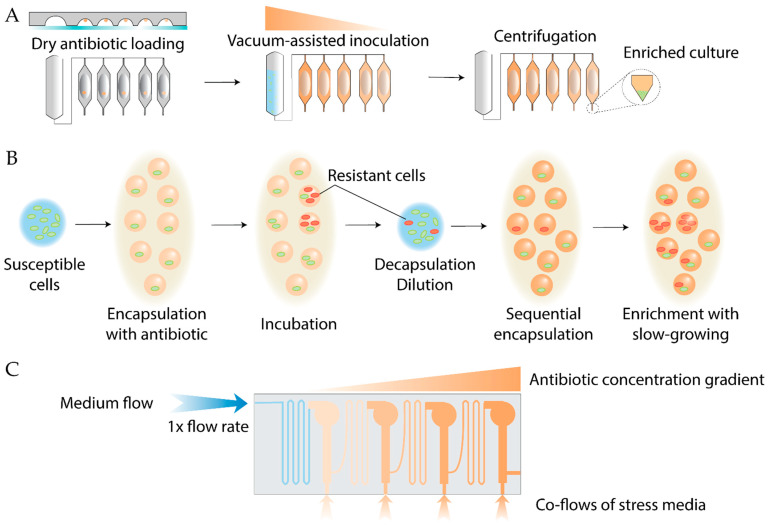
Experimental workflows to perform microfluidic-driven ALE. Devices and chips are PDMS replicas of SU-8/silicon master molds fabricated by photolithography. (**A**) Centrifugal microfluidic ALE system. (**B**) Droplet-based ALE. Emulsions are generated by flow-focusing in PDMS chips. (**C**) On-chip gradient ALE system.

Several microfluidic systems have been developed recently to enhance ALE efficiency (Figure 14). Standard ALE protocols are time-consuming; to accelerate resistance evolution, a centrifugal microfluidic system was designed [192]. In this device, a PDMS chip is pre-loaded with the antibiotic (with notably low sample consumption), bonded to glass, and subsequently filled with a bacterial suspension. On-chip centrifugation generates a condensed bacterial matrix, allowing survival under antibiotic concentrations several times higher than the MIC and promoting rapid resistance development (Figure 14A). Nonetheless, embedding antibiotics on PDMS prior to glass attachment requires chip assembly without plasma treatment, making the manufacturing process even more cumbersome than usual, significantly reducing the overall handling efficiency benefits.

A related on-chip approach employs spatially organized stress gradients to promote adaptation toward complex phenotypes (Figure 14C). This method enabled the identification of previously unrecognized mutations in *E. coli* conferring resistance to nalidixic acid [193]. Further studies on *E. coli* mutants resistant to ciprofloxacin generated in an on-chip spatial gradient revealed that the microfluidic environment itself can induce mutations beneficial for the subsequent evolution of highly resistant mutants [194]. Notably, shear-stress-induced mutations might also be considered as a limitation of on-chip ALE, because the misinterpretation of these genetic alterations can yield biased results. Evolution-on-a-chip platforms share common limitations, including proneness to clogging, cumbersome chip manufacturing and system operation, poor control over antibiotic absorption by PDMS, and a lack of standardization and commercially available systems, generally impairing the generalizability and reproducibility of the obtained results.

Conventional batch ALE methods are also limited by competitive exclusion, where slow-growing but strongly resistant variants are outcompeted, leading to diversity loss. Despite the reduced population size compared to batch cultivation, in separated populations, resistant mutants are more likely to become fixed [195]. Emulsion-based cultivation provides spatial isolation and fine control over environmental conditions, enabling the detection of distinct evolutionary trajectories—as shown for *P. aeruginosa* evolving resistance to colistin [196] and *E. coli* adaptation to doxycycline [197] (Figure 14B).

In addition to the identification of genetic determinants of resistance, microfluidic protocols can be applied to phenotyping mutant strains and detailed analysis of resistance gene dissemination processes by single-cell imaging (Table 3). For example, single-cell imaging of growth rate was applied to show the fitness cost of gene amplification-mediated resistance, demonstrating rapid loss of amplification in the absence of selection for clinical isolates of *E. coli* and *Salmonella enterica* [198]. Moreover, the observation of single colonies in the monolayer can be utilized for the detection of individual events of horizontal and vertical gene transfer [199,200]. The physiological role of known resistance-associated mutations can be elucidated on the basis of single-cell phenotyping. For instance, a previously described monolayer trap (Figure 12C) for *M. smegmatis* was utilized to compare persistent phenotypes in wild-type cells and the *msm2570::Tn* transposon mutant that has an insertion in the *msm2570* gene encoding a putative xanthine/uracil permease [201].

### 5.2. Phenotypic Resistance and Bacterial Stress Responses

Antibiotic therapy is never applied to a homogeneous, static target. Even within a single clonal culture, bacterial populations are dynamic, spatially structured, and phenotypically diverse. Studies that track how bacterial cultures respond to antibiotic exposure and how resistant subpopulations arise are, therefore, essential to understanding treatment success and failure. Single-cell resolution is extremely informative in a realm of non-heritable, phenotypic resistance studies. Mostly, these studies rely on classical single-cell imaging devices, described in Section 4.2.1 (Table 4).

Many recent studies aimed to characterize subpopulations of bacteria surviving drug exposure and elucidate key features underlying resistant phenotypes. Classical colony-forming unit (CFU)-based killing assays can misrepresent persister dynamics due to post-exposure killing, as demonstrated for *Salmonella* [202]. Tracking of the pre- and post-exposure history of persister formation was also successfully achieved for *E. coli* using a membrane-covered microchamber array (MCMA) [203]. These examples underline the greater biological relevance of microfluidics-based persister studies.

Phenotypic resistance is typically associated with growth-arrested persisters. Indeed, several studies revealed persister subpopulations exhibiting a dormant state of reduced growth rates. For example, a viable but non-cultural state was induced in *B. subtilis* prior to antibiotic exposure by osmotic stress, leading to antibiotic tolerance [204]. A fluorescent reporter for in vivo ATP measurements revealed that a subpopulations of *E. coli* with a low level of ATP survive ampicillin exposure, suggesting a “low-energy” mechanism of persistence [205].

Surprisingly, subpopulations of cells surviving the antibiotic exposure were found to exhibit diverse growth rates. Under ampicillin treatment, *E. coli* cells with ~50% reduced growth rate demonstrated lower antibiotic susceptibility compared to both normal and growth-arrested cells [206]. Moreover, a fast-growing macrolide-resistant subpopulation with high expression of ribosomal promoters was detected by investigation of cell-to-cell differences in drug uptake. Presumably, drug-free ribosomes facilitated essential cellular processes, including efflux of macrolides, leading to a fast-growing resistant phenotype [207]. Comparative studies across antibiotics revealed distinct stress-response mechanisms. One such study testing nine antibiotics shows that treatment with rifamycin and nitrofurantoin, differing in both structure and mode of action, led to surviving sub-populations [208]. Phenotypically resistant strains, particularly those with a high growth rate, significantly increase the risk of developing genetically fixed resistance.

Phenotypic resistance has diverse and elusive mechanisms, depending on a wide variety of factors. For example, studies on phenotypic resistance of *E. coli* showed that multicopy plasmids can serve as drivers of bacterial adaptation, enabling the rapid modulation of the number of gene copies [209]. Moreover, *E. coli* demonstrated physiological adaptation to antibiotic treatment after the deletion of resistance genes [210]. Phenotypic resistance to AMP can be mediated by efflux activity and was shown to be reverted by an efflux pump inhibitor [211]. Similar results were obtained for *S. aureus*, escaping β-lactam treatment by AbcA transporter overexpression [212]. Visualization of plasmid-encoded fluorescent pH reporter showed that *E. coli* persisters resistant to ampicillin have lower intracellular pH than viable but non-culturable and susceptible cells [213], highlighting that manipulation of intracellular pH can be a strategy for targeting persisters.

All the aforementioned studies applied fluorescent or optical imaging to monitor stressed populations. As a non-destructive and label-free alternative to imaging, Raman spectroscopy combined with acoustic trapping enabled monitoring of *M. smegmatis* response to antibiotic treatment [214]. Despite challenges in interpretation, Raman fingerprint monitoring is a promising approach to observing metabolic changes in live cells.

However, the most informative approach to assessing stress-induced metabolomics changes is mass spectrometry. On-chip lysis of antibiotic-treated bacteria with subsequent ESI-MS monitoring revealed the upregulation of the cyclic dinucleotide c-di-GMP in ESBL-producing *E. coli*. The treatment with a chemical inhibitor of c-di-GMP biosynthesis indeed reverted resistance to beta-lactams, verifying the identified self-saving response as a target for adjuvant development [215]. Despite obvious advantages, coupling microfluidic approaches with on-chip MS-detection is very technically challenging.

**Table 4:** Summary of experimental approaches to the study of heteroresistance.

Antibiotic	Device	Detection Method	Studied Strains	Observed Effect	Ref
Ampicillin Ciprofloxacin	MCMA	Microscopy	*E. coli*	Pre- and post-exposure imaging of individual cells	[203]
Ampicillin	Figure 11A MM	Microscopy	*E. coli*, expressing pHluorin	Intracellular pH in individual cells	[213]
Ampicillin	Figure 11A MM	Microscopy	*E. coli*, expressing iATPSnFr^1.0^	ATP levels in individual cells	[205]
Trimethoprim Linezolid Ciprofloxacin Roxithromycin Vancomycin Polymyxin Octapeptin Tachyplesin	Figure 11A MM	Microscopy	*E. coli* *P. aeruginosa* *Burkholderia cenocepacia* *S. aureus*	Accumulation of fluorescent antibiotic derivative	[207]
Chloramphenicol	Figure 11A MM	Microscopy	*E. coli*	Growth kinetics after resistance gene deletion	[210]
Chloramphenicol Gentamycin Spectinomycin Tetracycline Rifampicin Ciprofloxacin Nirtofurantoin Carbenicillin Ceftriaxone	Figure 11A MM	Microscopy	*E. coli*	Growth response under antibiotic treatment	[208]
Nafcillin Oxacillin	Figure 11A MM	Microscopy	*E.coli* with AbcA transporter overexpression	Individual cells growth rate	[212]
Tachyplesin (AMP)	Figure 11A MM	Microscopy	*E. coli* *P. aeruginosa*	Fluorescent antibiotic uptake	[211]
Ampicillin	Figure 11A MM	Microscopy	*E. coli*	Growth kinetics under antibiotic treatment	[206]
Flucloxacillin	Figure 12A	Microscopy	*Salmonella* *S. aureus*	Single-cell growth and regrowth kinetics	[202]
Kanamycin	Figure 12A	Microscopy	*B. subtilis*	Single-cell growth kinetics, fluorescent antibiotic uptake	[204]
Cefotaxime	Droplet (W/O)	Microscopy	*E. coli* expressing β-lactamases	Susceptibility distribution	[216]
Isoniazid	Acoustic trap	Raman spectroscopy	*M. smegmatis*	Single-cell metabolic response by Raman fingerprint	[214]
Ampicillin	Figure 12A	Microscopy	*E. coli*	Indirect monitoring of plasmid copy numbers	[209]
Ceftriaxone	HV-coupled channel	Mass-spectrometry	*E. coli*	Metabolic response to antibiotic treatment	[215]

In summary, recent imaging-based investigations reveal a diverse—and at times contradictory—landscape of phenotypic resistance. This variability reflects the inherently multifaceted nature of non-heritable tolerance. However, discrepancies between studies likely also stem from experimental biases introduced by microfluidic environments themselves. Factors such as geometric confinement, shear stress, nutrient gradients, droplet composition, dye toxicity, and material-dependent interactions can significantly modulate cellular physiology and influence the apparent abundance or behavior of tolerant subpopulations. Furthermore, differences in imaging modalities, detection thresholds, and data analysis pipelines add additional layers of variability. Taken together, these considerations underscore the importance of interpreting microfluidic single-cell data within the context of the experimental microenvironment and validating key observations using complementary approaches. As the field advances, standardization of microfluidic assays will be essential in studies of phenotypic resistance.

There are several biological questions in phenotypic resistance research that extend beyond standard single-cell imaging protocols. First, emerging studies highlight the role of biomolecular condensates in bacterial stress responses. Increasing evidence indicates that key cell cycle proteins form phase-separated condensates under stress, serving as adaptive organizational hubs and potential drug targets [217]. Droplet microfluidics has been used to model such condensates, providing mechanistic insight into *E. coli* division machinery [218]. Although the role of biomolecular condensates in antimicrobial tolerance is still poorly understood, they hold considerable potential for future microfluidic studies, which are uniquely suited for fine-tuning environmental conditions and quantifying protein phase transitions in a controlled manner.

**Figure 15:**
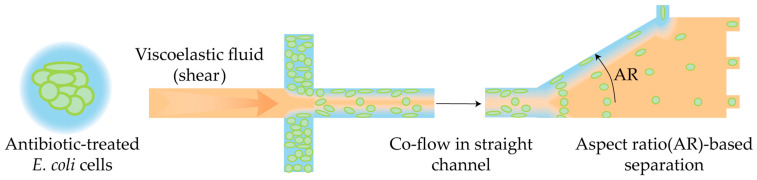
Viscoelastic shape-based separation of *E. coli* subpopulations. The microfluidic device is a PDMS replica of a SU-8/silicon master mold fabricated by photolithography.

Second, whereas most phenotyping studies rely on qualitative descriptions and rarely pursue isolation of specific microcolonies or subpopulations, downstream manipulation and recovery of specific phenotypes can be highly informative. In microfluidic assays, recovery of specific cell states typically requires active sorting (e.g., FACS or FADS). Recently, however, passive sorting techniques have gained attention as scalable, low-cost alternatives [18]. One of the approaches to passive sorting is vascoelasting microfluidics, which has recently been successfully adapted for shape-based separation of β-lactam-treated *E. coli* cells [219]. The device (Figure 15) integrates two inlets for viscoelastic and Newtonian fluids and seven outlets; spherical bacteria migrate toward the center of the channel, while elongated cells are directed to the sides, enabling label-free morphological separation.

### 5.3. Chemotaxis and Bacterial Motility

**Figure 16:**
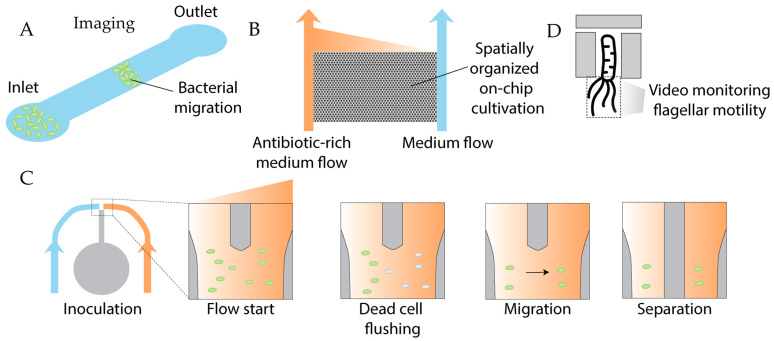
Microfluidic models for the study of spatial gradients and bacterial motility. (**A**) Bacterial migration channel. The PDMS stamp is produced by replication from a deep reactive ion etching-fabricated silicon master; an optical adhesive layer is then transferred by stamping and is bonded between glass and PMMA substrates. (**B**) Bacterial cultivation under controlled chemical gradients. The chip is a PDMS replica of a DRIE-patterned silicon master. Bright-field microscopy is used for imaging. (**C**) Microfluidic circuits for isolating cells of interest after migration. The device is fabricated via 3D printing. (**D**) Single-cell motility assay. The chips are PDMS replicas of SU-8/silicon master molds fabricated by photolithography.

Microfluidic models can reproduce spatial gradients of antibiotics and nutrients, reflecting environmental and host conditions (Figure 16). For example, studies of *P. aeruginosa* in long gradient channels revealed that flow-shaped gradients significantly alter antibiotic susceptibility [220]. Several approaches to the study of bacterial chemotaxis were applied recently, including the imaging of *E. coli* migration in a bacterial monolayer [221] (Figure 16A), *Shewanella oneidensis* chemotaxis in a microfluidic gradient chamber [222], *E. coli* growth in a spatially controlled population under tetracycline treatment (Figure 16B), and suicidal chemotaxis of *P. aeruginosa* towards high antibiotic concentrations [223,224]. The latter effect was studied in detail using a reconfigurable microfluidic device, allowing for the isolation of subpopulations after time-lapse imaging by building fluid walls around migrating cells (Figure 16C).

A complementary “static” strategy uses hydrodynamic trapping of individual bacteria followed by high-speed video microscopy (Figure 16D). As demonstrated on *E. coli* MG1655 and *Salmonella typhimurium*, reduced single-cell motility under kanamycin exposure correlates with growth inhibition and can serve as a proxy for heteroresistance [225].

### 5.4. Antibiotic Susceptibility of Biofilms

As discussed in Section 3, biofilms represent highly organized microbial communities that are inherently more resistant to antibiotics than planktonic cells. Moreover, they act as hotspots for horizontal gene transfer and resistance dissemination. Microfluidic platforms, including flow chambers and the BioFlux system (Figure 10A), are widely used to study biofilm-associated resistance. For instance, flow-chamber models were employed to identify *P. aeruginosa* biofilm resistance genes [226] and to investigate the role of the flagellar hook in a biofilm structure [227]. The BioFlux platform, combined with fluorescence microscopy, revealed a strong correlation between biofilm formation and clarithromycin resistance in clinical *Helicobacter pylori* isolates [228].

**Figure 17:**
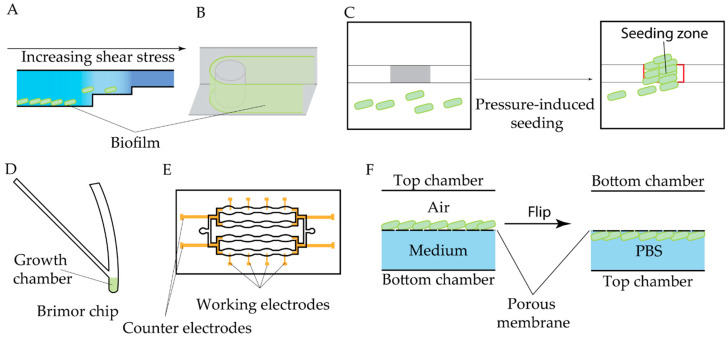
Microfluidic biofilm models for precise monitoring of stress responses. (**A**) Channels for shear stress variation. Chips are PDMS replicas of SU-8/silicon master molds fabricated by photolithography. (**B**) Pillar-induced streamer formation. Chips are PDMS replicas of SU-8/silicon master molds. Biofilm streamers are visualized by fluorescence microscopy. (**C**) Pressure-controlled biofilm seeding. Chips are PDMS replicas of AZ5462/silicon master molds fabricated by photolithography and bonded to glass slides. (**D**) The Brimor chip. Devices are PDMS replicas of SU-8/silicon master molds. (**E**) Lego-like microfluidic biofilm-on-chip platform. Chips are PDMS replicas of DRIE-patterned silicon masters. Biofilms are monitored by impedance measurements. (**F**) Air–liquid interface biofilm formation device. The chip consists of a PDMS replica of a 3D-printed master combined with a polyester membrane and 3D-printed covers with integrated carbon electrodes. Biofilm development is analyzed by impedance.

Fluid shear stress is another key factor influencing biofilm morphology and antibiotic susceptibility. For example, clinical isolates of *Klebsiella pneumoniae* exposed to shear stress in microfluidic channels showed increased viability under antibiotic treatment and enhanced proliferation within macrophages [229]. To simultaneously evaluate shear stress and antibiotic exposure, a double-layer microfluidic biofilm chip (2PAB) was developed, featuring a concentration gradient generator (tree-shaped, Figure 4A) and expanding flow chambers (Figure 17A) [230]. Studies using this system demonstrated species-specific differences in *E. coli* and *P. aeruginosa* biofilm eradication, emphasizing the need for controlled hydrodynamic environments.

Geometric design can strongly modulate biofilm behavior. Wavy channel geometry can create heterogeneous flow conditions, promoting localized regions of low shear stress [231] (Figure 17E). The introduction of an isolated pillar into a straight channel, located at its half-width, induced reproducible biofilm streamer–filament formations suspended in the flow [232] (Figure 17B). Using this platform, antibiotic-induced genotoxic stress and SOS-response were shown to cause explosive cell lysis and stimulate streamer formation in the opportunistic pathogen *Burkholderia cenocepacia* [233].

To overcome the limitations of standard flow-chamber models—such as random adhesion, clogging, or limited harvesting—new systems have been developed (Table 5). A spatially controlled seeding chip with a pressure-based seeding gap (Figure 17C) allows for reproducible biofilm formation [234]. A microfluidic chip called Brimor was developed to study the enrichment dynamics of antibiotic-resistant bacteria in biofilms using confocal microscopy [235]. The Brimor chip is manufactured from a 3D-printed mold and includes two channels for the simultaneous observation of experimental and control biofilms (Figure 17D). Modular chips integrating electrodes have been introduced for monitoring biofilm migration and regrowth dynamics (Figure 17E) [231]. Finally, a dual-chamber microfluidic device was designed to model *P. aeruginosa* biofilm formation at the air–liquid interface, with electrochemical detection of pyocyanin serving as a proxy for viable cell density [236] (Figure 17F).

To conclude, investigations of resistance development in biofilms have driven the emergence of increasingly sophisticated biofilm-on-chip models that go well beyond simple flow chambers. These systems enable a controlled variation in shear stress, initial seeding patterns, oxygen permeation, and air–liquid interface modeling. However, despite these advantages, current microfluidic biofilm platforms still capture only a subset of the biological and environmental complexity that governs biofilm-associated antibiotic tolerance in real settings. Material-dependent artifacts—such as antibiotic adsorption to PDMS, altered oxygen availability, or shear-induced physiological changes—can further bias results and complicate quantitative interpretation. As a result, even sophisticated microfluidic models may underestimate the resilience of clinically relevant biofilms or misrepresent the mechanisms underlying treatment failure.

Moreover, most available systems address only isolated subsets of the relevant limitations, underscoring the need for standardized and validated biofilm models that integrate the most successful design features into a unified framework. Continued progress will depend on developing physiologically informed platforms that incorporate polymicrobial communities, host-mimicking microenvironments, and more realistic mechanical and chemical gradients, coupled with rigorous benchmarking against in vivo or ex vivo reference systems.

## 6. Conclusions and Future Directions

Microfluidic technologies have reshaped the landscape of antibiotic resistance research by enabling precise, high-resolution observation of microbial dynamics under controlled conditions. The versatility of these systems allows them to bridge the gap between molecular-scale mechanisms and population-level phenomena, providing insights that are often inaccessible through traditional methods. Recent advancements demonstrate that microfluidics can be applied across the full spectrum of antimicrobial studies—from the discovery of new antibiotic producers and the screening of natural or synthetic compounds to the mechanistic dissection of drug action and quantitative analysis of stress responses and resistance evolution. Whereas specific limitations and development directions are discussed in specialized sections of this review, here we summarize the general challenges and the possible solutions to them.

It is essential to acknowledge that the microenvironment in a microfluidic device can introduce biases that influence experimental outcomes. These artifacts do not invalidate microfluidic studies but must be recognized, controlled, and appropriately contextualized, particularly when drawing biological conclusions or comparing results with conventional assays. First, device materials can significantly shape experimental conditions. PDMS, still the most commonly used microfluidic material, absorbs hydrophobic antibiotics, membrane-active compounds, fluorescent dyes, and even quorum-sensing molecules, altering experimental conditions in a poorly controlled manner and limiting chemical compatibility. PDMS is also gas-permeable, requiring additional manipulations to achieve anaerobic conditions. Second, chip design and geometric confinement can shift microbial physiology in ways that are difficult to replicate in bulk systems. These confinement effects can bias measurements of growth rates, persistence, drug penetration, or stress signaling.

Limited standardization of device designs and protocols also continues to hinder data comparability and large-scale implementation. Traditionally, many emerging assays are introduced using custom designs of monolithic chips or highly integrated devices. Although the field has produced numerous elegant and technically advanced devices, the overwhelming majority remain proof-of-concept demonstrations that fail to transition into robust or commercially viable platforms [237]. By contrast, our survey of the recent literature illustrates that the most widely adopted platforms, such as BioFlux or simple monolayer cell traps, are not the most technologically sophisticated but the most operationally tractable. A recurring theme in the literature is the misalignment between device innovation and biological relevance. This suggests that future microfluidic development should prioritize ease of use, modularity, and long-term device applicability over maximizing integration or novelty. Some of the challenges can be mitigated through the development of standards and guidelines in microfluidics, which can enable reliable and validated data acquisition and streamline manufacturing processes [238]. Implementation of robust, well-characterized commercially available modules can also enhance reproducibility of the obtained results [239,240]. Compatibility with existing laboratory infrastructure is also important: droplet microfluidics, which integrates with fluorescence microscopy and FACS, exemplifies how alignment with standard equipment enables widespread use.

Another critical challenge concerns data analysis. Most current microfluidics protocols in antimicrobial resistance studies, summarized in this review, rely on image analysis to some extent. Time-lapse imaging, fluorescent barcoding, and droplet indexing generate large, complex datasets that quickly overwhelm manual analysis workflows. While microfluidics is generally oriented toward high-throughput analysis, data analysis creates a bottleneck for further development of the field. Emerging AI-assisted approaches are reshaping data processing and analysis; this field is rapidly growing [6,241,242]. One of the most promising applications is the development of unsupervised machine learning models for label-free detection and phenotypic classification. Despite proven synergistic acceleration in microfluidics from AI implementation, these methods also introduce new bottlenecks. Training deep learning models requires substantial technical expertise, large annotated training datasets, stable data acquisition pipelines, and considerable computational resources. Furthermore, the “black box” nature of modern AI approaches can limit interpretability [243]; in addition, model training and parameter tuning can introduce biases. Nonetheless, further AI implementation in a microfluidics system offers very intriguing opportunities, including (1) multimodal detection and multi-omics data synthesis for the development of more informative assays; (2) adaptation of AI for informed droplet sorting and manipulation; (3) implementation of machine learning in device design [244].

Microfluidic technologies have found applications in a wide variety of research areas and continue to evolve rapidly. Each study addressing a specific scientific problem often introduces technical innovations with potential utility in other, even distant, disciplines. Furthermore, many works focus on improving encapsulation and imaging methods without direct applications to particular biological problems. For instance, recent studies have proposed enhanced image analysis algorithms for MM experiments [245]. Although such developments hold great promise, identifying their relevance to specialized research domains can be challenging, emphasizing the importance of comprehensive literature analyses and reviews summarizing emerging applications of microfluidics technologies.

The most rapidly advancing fields of microfluidics research aimed at combating antibiotic resistance include pathogen detection, antimicrobial susceptibility testing, and rapid drug selection in clinical settings. Studies in these areas mostly focus on simplifying device architecture, reducing analysis time, and enabling measurements at low bacterial concentrations within complex biological matrices. Nevertheless, many of these developments also hold significant potential for fundamental microbiological research. For example, the application of SlipChip technology for antimicrobial susceptibility testing (AST) [246,247,248] has evolved into dedicated platforms for novel antibiotic [146] and phage discovery [157]. Also, alternative methods for bacterial growth detection and live/dead sorting are continuously emerging and being refined, including those based on optofluidics [249,250], light scattering [251], Raman spectroscopy [252,253], impedance sensing [254], the di-electrophoresis effect [255], THz detection [256], and fluorescence measurements [257]. The development of systems for laboratory antibiotic resistance studies and integrating these detection approaches, especially when combining them using the aforementioned automated, AI-assisted multimodal data analysis systems, can pave the way for a qualitatively deeper understanding of bacterial physiology.

As microfluidics technologies continue to mature, they are tending towards becoming indispensable as tools in antimicrobial research, supporting natural compound discovery, rational drug design, phenotypic screening, and real-time resistance monitoring. Ultimately, the convergence of microfluidics with systems biology, synthetic biology, and computational analysis holds the potential to redefine our understanding of microbial adaptation and guide the next generation of strategies to overcome antibiotic resistance.

## Figures and Tables

**Table 3:** Recent single-cell studies on genetic bacterial resistance.

Antibiotic	Device	Detection Method	Studied Strains	Observed Effect	Ref
MDR strains	Figure 11A MM	Microscopy	*E. coli* *Salmonella enterica*	Single-cell growth rate	[198]
β-lactams	Figure 11A	Microscopy	*E. coli*	Visualization of conjugational and vertical transfer events	[199]
Isoniazid	Figure 12C	Microscopy	*M. smegmatis* *msm2570::Tn* mutant	Individual cell growth and lysis kinetics	[201]

**Table 5:** Summary of recently introduced microfluidic models of biofilm eradication.

Antibiotics	Device	Detection Method	Tested Biofilms	Key Features	Ref
Gentamicin Streptomycin	2PAB Figure 17A	Microscopy	*E. coli* *P. aeruginosa*	Simultaneous control of antibiotic treatment and shear stress	[230]
Berberine	Figure 17C	Microscopy	*E. coli* *P. aeruginosa* *S. typimurium* *K. pneumonia* *B. subtilis* *S. aureus* *E. faecium* *M. smegmatis*	Spatially controlled seeding Epoxy-resin sealing to block oxygen penetration Suitable for broad range of bacteria	[234]
Ciprofloxacin	Brimor Figure 17D	Microscopy	*E. coli*	Long-term cultivation Simple manufacturing procedure	[235]
Ciprofloxacin	Figure 17F	Pyocyanin detection	*P. aeruginosa*	Air–liquid interface biofilms Electrochemical detection of biomarker	[236]
Tetracycline Chloramphenicol Amikacin Coatings and nanoparticles	Figure 17E	Microscopy, electrical impedance	*P. aeruginosa*	Label-free monitoring Modular structure to assess migration and regrowth Localized shear stress variations	[231]
Mitomycin C Ciprofloxacin	Figure 17B	Microscopy	*P. aeruginosa* *Burkholderia cenocepacia*	Visualization of streamers	[232,233]

## Data Availability

Not applicable.
